# Novel Natural Compounds and Their Anatomical Distribution in the Stinging Fireworm *Hermodice carunculata* (Annelida)

**DOI:** 10.3390/md20090585

**Published:** 2022-09-19

**Authors:** Sara Righi, Luca Forti, Roberto Simonini, Valentina Ferrari, Daniela Prevedelli, Adele Mucci

**Affiliations:** 1 Department of Life Sciences, University of Modena and Reggio Emilia, Via Campi 213/D, 41125 Modena, Italy; 2 Department of Life Sciences, University of Modena and Reggio Emilia, Via Campi 103, 41125 Modena, Italy; 3 Department of Chemical and Geological Sciences, University of Modena and Reggio Emilia, Via Campi 103, 41125 Modena, Italy

**Keywords:** marine natural products, amphinomid, chemical defences, aposematism, phylogeny

## Abstract

Increasing evidence in the field of bioprospection fosters the necessity of studying poorly investigated poisonous marine invertebrates to expand knowledge on animal venom biology. Among marine annelids, amphinomid fireworms are notorious for their bearded trunk equipped with a powerful stinging capacity. Here, a methodological workflow based on analytical chemistry techniques (compound isolation followed by mass spectrometry and spectroscopy analyses) was applied to gain new insights, leading to the identification and structural elucidation of an array of natural products from Mediterranean specimens of *Hermodice carunculata*. Eight betaine-derived unprecedented compounds, named “carunculines”, were detected, bearing two terminal ammonium groups tri-and disubstituted at the Cα (A, B) and a series of different alkyl chains (I–VIII). The mixture of chemicals was found in all the body parts of *H. carunculata*, supporting a mechanism of action triggered by their vehiculation inside the dorsal chaetae, and subsequent injection when chaetae break off on contact. Preliminary investigations to understand adaptive features were also performed, showing a trend in carunculine abundance that fits into the evolutionary history of these worms. These findings shed light on the chemical ecology of amphinomids, giving reasons for the success of *H. carunculata* in benthic environments and providing promising novel metabolites for biotechnological implications.

## 1. Introduction

Natural products constitute a complex mixture of “secondary metabolites” not implicated in primary metabolic pathways, such as growth and development [1,2,3]. They provide the host organism with adaptive advantages, such as those related to anti-predatory weapons, symbioses, competition, reproduction and larval settlement [4].

Considering the wide range of possible intra- and interspecific interactions, Marine Natural Products (hereinafter “MNPs”) show an enormous diversity, which evolved to enable the organisms that produce them to survive and thrive [5,6,7]. Most MNPs have been discovered in sponges, cnidarians and nudibranch mollusks, and there is still a broad range of marine invertebrates whose arsenal of natural products remains poorly investigated.

Although the phylum Annelida constitutes the dominant benthic macrofauna from the intertidal zone down to the deep sea vents [8], far fewer of its chemicals have been characterized than those of other marine invertebrates. Indeed, no secondary metabolite from marine annelids has been reported in annual reviews on MNPs in general [9]. Chemicals including halogenated aromatics, proteins, amino acids and lumazine derivatives were mostly found in the families Sabellidae, Terebellidae, Glyceridae, and Nereididae [10]. Furthermore, “thelepamide” and “nebulosin” have recently been isolated and structurally characterized from *Thelepus crispus* and *Eupolymnia nebulosa* respectively [11,12], but chemical defenses from mobile polychaetes remain largely unexplored, and their ecological role has yet to be demonstrated [13,14].

Glycerids (bloodworms) and amphinomids (fireworms) have attracted attention in the field of marine envenomation due to their harmful interactions with fishermen and bathers. Bloodworms are equipped with four strong jaws that inject a proteinaceous venom [15,16], while fireworms display stinging dorsal chaetae. In the largest and most charismatic species, the bearded fireworm *Hermodice carunculata*, the dorsal chaetae break off when touched, playing both defensive and offensive actions against predators and preys, and inducing cutaneous inflammation in people affected [17,18]. The high sensitivity to breakage on contact of the chaetae might be ascribed to their calcareous nature and inner structure, characterized by a central cavity that could store chemicals [19]. Indeed, close examinations of predator-prey interactions and feeding bioassays have provided evidence for the deterrent action of the dorsal chaetae, which is triggered by a synergy between mechanical penetration and the release of compounds [18].

**Figure 1:**
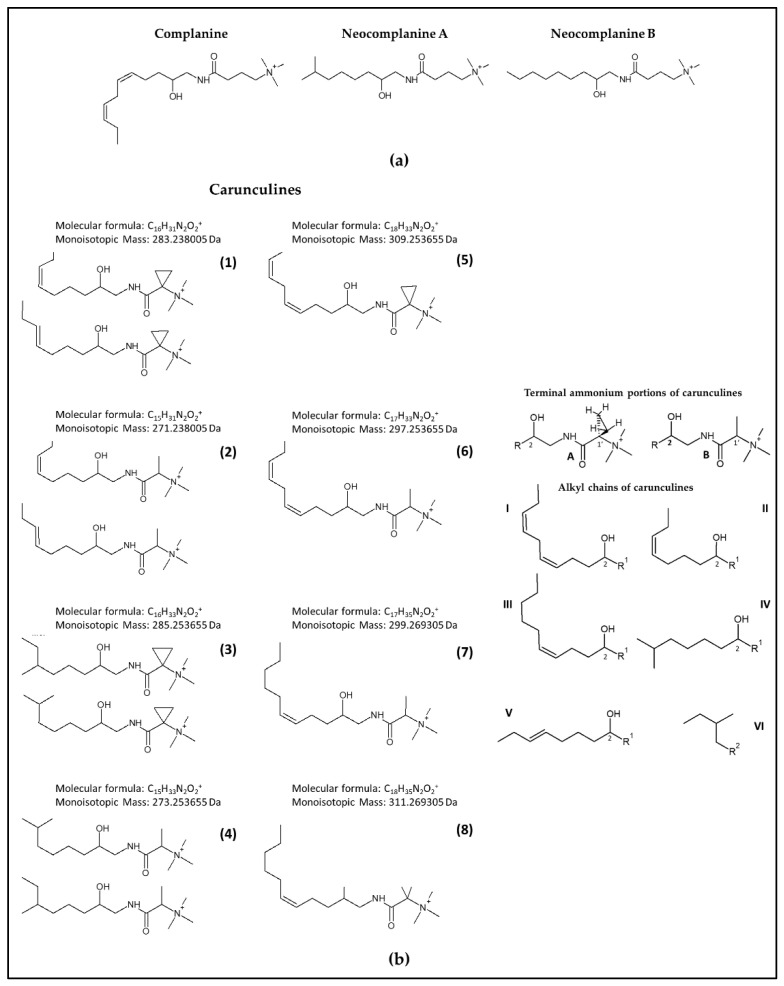
Molecular structures of complanines and hypotheses for carunculines. (**a**) Molecular structures of complanine and neocomplanines [20,21]. (**b**) Left panel: hypothesized molecular structures for carunculines (**1**–**8**) and their isomers derived by matching the structures obtained by NMR spectra and the formulae obtained by HPLC-ESI/HRMS data. R = alkyl chain I–VI; R1 = terminal ammonium portion (A) or (B); R2 = probably –(CH_2_)_2_–CHOH–R1. C2 are superimposed in the final structures. Right panel: terminal ammonium groups (A) and (B) and types of alkyl chains (I–VI) derived by NMR analysis.

To date, the only acute inflammation inducer that has been identified from an amphinomid is a trimethylammonium compound named “complanine” (Figure 1a), an amino alcohol isolated from the fireworm *Eurythoe complanata* [20]. Together with complanine, other analogs have been found and chemically synthesized to elucidate their structure (i.e., neocomplanines, Figure 1a) [21], but the ecological role of these chemicals and their distribution within the worm remain uninvestigated.

Given the high phylogenetic closeness of *E. complanata* to *H. carunculata* [22], we hypothesized that secondary metabolites related to complanine could also account for the stinging capacity of the latter species [18]. Quite surprisingly, during our efforts to isolate the amino alcohol, we found an unexpected chemical diversity of metabolites whose structures differed from complanine and that underpin fireworm stinging capacity as a whole.

In this paper, we describe the occurrence of eight novel quaternary ammonium compounds, named carunculines (**1**–**8**), in Mediterranean *H. carunculata*. These molecules, with different carbon chain lengths and unsaturation degrees, are all characterized by a linear or methyl substituted alkyl amino alcohol structure, like several related compounds from tunicates, sponges and clams [23,24,25,26,27,28,29]. We decided to focus on the chemical profile of the overall mixture, rather than on single compounds, to investigate their anatomical distribution in order to assess whether selective storage occurs. Considering the palatability of fireworm body parts and the powerful offensive action of the notochaetae [17,18], we expected to find carunculines in the latter. A range of representative marine invertebrate taxa, including annelids belonging to families other than amphinomids, was also screened to infer the presence of these chemicals in different lineages and their potential ecological roles in an evolutionary context. It was our belief that carunculines do not constitute the distinctive secondary metabolites of other non-urticating marine invertebrates.

## 2. Results

### 2.1. Identification of Novel Compounds by Mass Spectrometry and NMR Spectroscopy

The fraction partitioned in MeOH/H_2_O of the acetone extract from specimens of *H. carunculata* collected in Apulia (Italy) was purified testing different solvents and gradients. The purified mixture of target compounds was then analysed through mass spectrometry (using an HPLC-ESI/HRMS) and NMR spectroscopy.

All the mobile phases tested in HPLC-ESI/HRMS enabled the elution of carunculines. The mass spectra provided eight main molecular ions with related isomers, which were eluted in the retention time (RT) range 4.1–6.4 min (see [18] as reference) (Figure S1, Supplementary Materials).

However, carunculine elution often occurred in different fractions during the column chromatography, together with other non-target compounds. Even though different mixtures of organo-halogenated solvents were successful for elution purposes (**1**–**8**) (Table S1, Supplementary Materials), the most effective was the one employing a gradient of ethyl acetate/methanol (EtOAc/MeOH) ending with acidified water: (1) EtOAc-MeOH (3:2), (2) EtOAc-MeOH (2:3), (3) EtOAc-MeOH (1:4), (4) MeOH, (5) H_2_O, (6) H_2_O + 0.4% acetic acid (AcOH). In particular, the Full MS chromatograms of the fraction n. 6 (i.e., eluted by H_2_O + 0.4% AcOH) consisted of a purified mixture of carunculines **1**–**8**, and 43.9 mg were obtained starting from 71 g of dried fireworms (Table S1, Supplementary Materials). The identification of the target compounds was then confirmed by in-depth analyses of this fraction.

The molecular formulae of carunculines (**1**–**8**) were derived using HPLC-ESI/HRMS from the molecular ions at *m*/*z* 283.2377 (C_16_H_31_N_2_O_2_^+^) (**1**), *m*/*z* 271.2377 (C_15_H_31_N_2_O_2_^+^) (**2**), *m*/*z* 285.2535 (C_16_H_33_N_2_O_2_^+^) (**3**), *m*/*z* 273.2535 (C_15_H_33_N_2_O_2_^+^) (**4**), *m*/*z* 309.2535 (C_18_H_33_N_2_O_2_^+^) (**5**), *m*/*z* 297.2535 (C_17_H_33_N_2_O_2_^+^) (**6**), *m*/*z* 299.2693 (C_17_H_35_N_2_O_2_^+^) (**7**) and *m*/*z* 311.2693 (C_18_H_35_N_2_O_2_^+^) (8) (Figure 1). The molecular formulae indicated three degrees of unsaturation for carunculines **1**, **6**, **8**, two degrees of unsaturation for carunculines **2**, **3**, **7**, four degrees of unsaturation for carunculine **5**, and one degree of unsaturation for carunculine **4** (Figure 1b).

The occurrence of different metabolites and related isomers was further supported by 1D and 2D NMR analyses, which revealed a complex mixture of both saturated and unsaturated amino alcohols.

The structures of carunculines **1**–**8** were hypothesized (Figure 1b), matching the molecular structures derived by NMR assignments (Figure 2 and Figures S2–S9, Supplementary Materials), and the exact masses provided by HPLC-ESI/HRMS (Figure 3a).

NMR data enabled the reconstruction of the molecular structure, on the basis of H,N correlations, one-bond (HSQCed) and multiple-bond (HMBC) H,C correlations, through-bond (COSY, TOCSY), through-space (NOESY) H,H correlations and HSQC-TOCSY H,C correlations. The detailed analysis of spectroscopic data disclosed the structure of prominent compounds, characterized by two types of trimethylammonium groups different from those of complanine. Indeed, the former of these novel units (A, the most abundant) is characterized by the presence of a cyclopropyl ring bound on C1′ to the amide carbonyl and to the ammonium group, whereas the latter (B, the less abundant) features a methyl group on C1′, as reported in Figure 1b. In terms of compositions, these two moieties differ by one carbon and two hydrogen atoms from each other. In addition, six different alkyl chains (I–VI) were identified (Figure 1b).

The starting point for the structural assignment were the NCH_3_^+^ proton and carbon signals that are recognized by their shape (singlets) and characteristic chemical shifts. We found two major singlets at 3.23/53.8 (higher) and 3.21/52.6 ppm (lower) in the H,C-HSQCed spectrum (Figure S3). From the H,N-HSQC experiment we derived the two H,N correlations at 3.23/49.8 (higher) and 3.21/52.1 ppm (lower) (Figure S4). Other H,N correlations with the same nitrogens are also detected at around 1.76/49.8 and 1.38/49.8 (lower in intensity) and at around 1.63/52.1 ppm, this last given by two overlapped doublets in the proton dimension. The doublets at 1.63 ppm (*J* = 6.9 Hz) correlate in the COSY spectrum (Figure S5) with CHs around 4.06/71.4 ppm (from H,C-HSQCed). The two groups of signals around 1.76/49.8 and 1.38/49.8 were, at a first analysis, attributed to couples of diastereotopic CH_3_ (from H,C-HSQCed) that correlate with quaternary carbons around 60.0 ppm in the H,C-HMBC spectrum (Figure S6). Nevertheless, they showed too high correlations between each other in the COSY spectrum, and the pattern of these set of correlations was particularly odd (Figure S5). At the same time, the phase of the H,C-correlations in the HSQCed spectrum could not be properly adjusted. All these observations suggested a second hypothesis to us: the presence of a substituted cyclopropane ring. In fact, the HSQCed experiment works well when ^1^JCH increases with chemical shifts. Proton and carbon NMR signals of three-term rings, such as cyclopropanes and epoxides, are found in the aliphatic region but are characterized by ^1^JCH (160 and 175 Hz, respectively) higher than those of common aliphatic (125–145 Hz) H,C pairs. This causes the signs of H,C correlations in HSQCed spectra of three term cycles to be misleading. Once correctly attributed these signals to a 1,1-disubstituted cyclopropane ring, their shape becomes directly interpretable as due to an AA’BB’ spin system: two symmetric second order multiplets centered at 1.76 and 1.38 ppm, due to the two couples of diastereotopic methylene protons (Figure S5).

These methylene signals also correlate with carbonyl carbons at 167.2 ppm, whereas the methyl doublets at 1.63 ppm correlates with a carbonyl at 168.8 ppm in the H,C-HMBC spectrum (Figure S6). These sets of correlations allow us to reconstruct the two α-acyl ammonium groups reported in Figure 1 as (A) and (B). At least two carunculines of type (B) are recognized by the presence of two overlapped doublets around 1.63 ppm.

The carbonyls in moieties (A) and (B) are part of the amide groups, as derived by long range correlations with two types of methylenes around 3.33,3.26/46.2 and 3.36,3.27/45.6 ppm (Figure S6). Both these methylene protons correlate with two groups of CHOH around 3.76/70.2 and 3.76/69.8 ppm. These oxygenated methine carbons show different correlation sets in the HSQC-TOCSY spectrum. The carbon signals around 69.8 ppm allow to detect 5.45, 2.83, 2.17, 1.51 and 0.96 ppm proton signals, whereas carbons around 70.2 ppm correlate with 5.49, 5.40, 2.08, 1.48, 1.44, 1.40, 1.31 and 1.17 ppm (low). These two pools of resonances derive from overlapped signals of saturated and unsaturated alkyl chains. We tried to disentangle them through the analysis of COSY, TOCSY, HSQCed, HMBC, HSQC-TOCSY and NOESY experiments (Figures S3–S9, Supplementary Materials). The reconstructed chain structures are collected in Figure 1 and the chemical shift assignments are found in Figure S2, Supplementary Materials.

**Figure 3:**
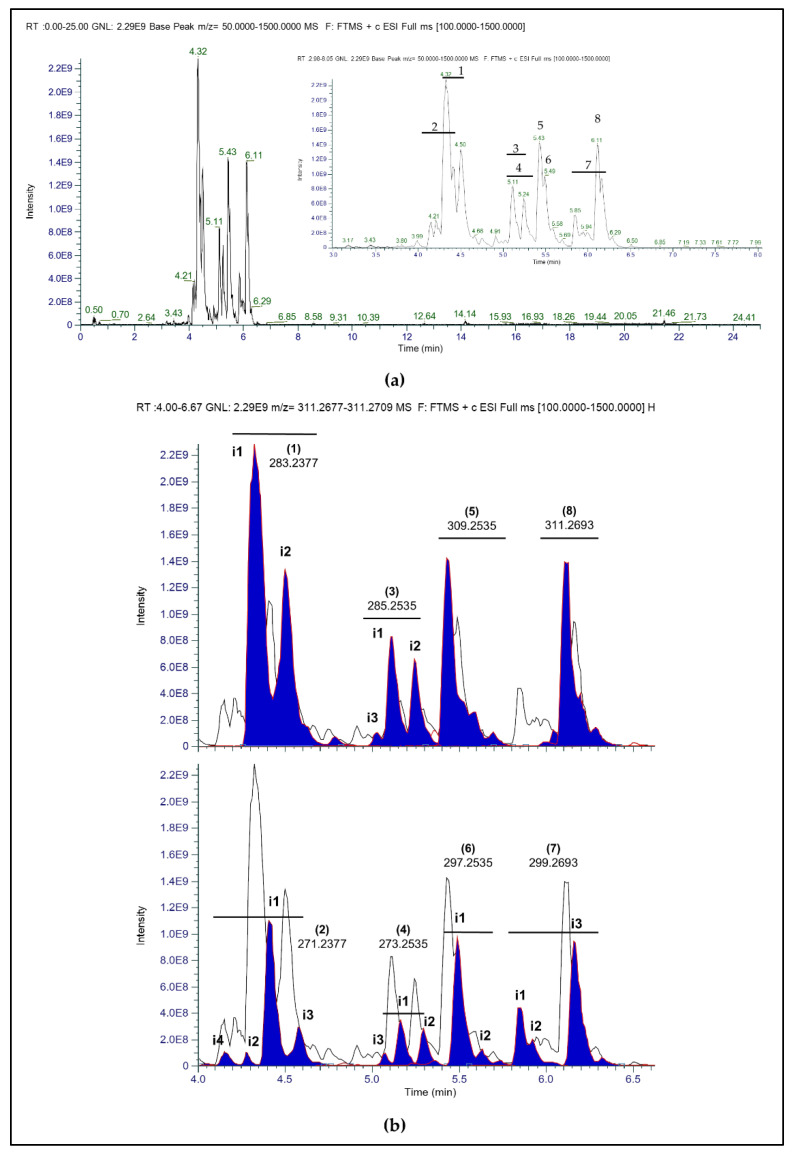
HPLC-ESI/HRMS chromatograms of carunculines (**1**–**8**) eluted by H_2_O + 0.4% AcOH. (**a**) Full MS chromatogram of the fraction with a magnified view of the target compounds (on the right). (**b**) Extracted ion chromatogram of carunculines (**1**–**8**) from *H. carunculata*: the peaks of compounds **1**,**3**,**5**,**8** (up) and **2**,**4**,**6**,**7** (down) and related isomers (i1,i2,i3,i4) are filled in blue, respectively.

Regarding HPLC-ESI/HRMS analyses of carunculines, the QuanBrowser software enabled the detection of two isomers for **1** and **6**, and four isomers for **2**, while **3**, **4** and **7** displayed three isomers, and only one occurred for **5** and **8** (Figure 3b).

Attempts to determine the structure of carunculine **1**, with molecular formula C_16_H_31_N_2_O_2_^+^, suggested the presence of a terminal glycine betaine, with the Cα incorporated in a cyclopropane ring (A), and the alkyl chain C_8_H_15_O with one double bond at C6 of II and V type (Figure 1b).

Carunculine **2** has the molecular formula C_15_H_31_N_2_O_2_^+^ and appears to be composed of an alkyl chain C_10_H_19_O with a double bond at C6 (II and V type), and the terminal ammonium portion (B), derived from alanine betaine, for a total of four possible isomers.

The mass spectrum of carunculine **3** provided the molecular formula C_16_H_33_N_2_O_2_. NMR spectra supported the presence of at least two different saturated hydrocarbon chains (C_8_H_17_O). The assembly of these chains with (A) is coherent with the presence of three isomers (two of which diastereomers), as found in the ion chromatogram reported in Figure 3b.

The structure suggested for carunculine **4** (C_15_H_33_N_2_O_2_^+^) combining HPLC-ESI/HRMS and NMR data is obtained matching the (B)-type terminal ammonium group with saturated chains IV and VI (C_8_H_17_O). In this case, two main isomers of the six possible were detected.

The structure of carunculine **5** seems to be characterized by (A) in the terminal ammonium portion and the I-type structure of the alkyl chain.

The NMR data supported close similarity between carunculines **6** and **7**, with molecular formulae of C_17_H_33_N_2_O_2_^+^ and C_17_H_35_N_2_O_2_^+^, respectively, as established by HPLC-ESI/HRMS. These molecules share the terminal ammonium portion (B) and present the different alkyl chains I and III, with two and one double bonds, respectively, at C5 and/or C8. Three isomers were detected for carunculine **7**, implying a possible isomerization of the double bond, which however was not clearly evidenced by NMR data, and could account for the minor isomer (Figure 3b).

The only isomer of carunculine **8** was identified by matching the (A)-type ammonium termination with the hydrocarbon chain with a double bond in C5 (III).

Further MS/MS analyses using HPLC-ESI/HRMS highlighted the regular presence of different fragments, coherent with the structures derived by the NMR data. In particular, fragments ascribable to ions derived from the ammonium group (CH_2_ = N(CH_3_)_2_^+^, *m*/*z* 58.0659; NH(CH_3_)_3_^+^, *m*/*z* 60.0815), from the alkyl chains (C_5_H_7_^+^, *m*/*z* 67.0549, C_7_H_11_^+^, *m*/*z* 95.0859, C_9_H_15_^+^, *m*/*z* 123.1169) as well as from the loss of one water molecule [M − H_2_O]^+^ were always detected. In carunculines **1**,**3**,**5**,**8** characterized by the (A)-type termination, common fragments were also *m*/*z* 116.1071 and *m*/*z* 143.1178, and the losses of neutrals corresponding to *m* 85.0528 and *m* 113.0843 (see Table S2, Supplementary Materials, for the hypothesized structures). The mass fragmentation spectra of carunculines with the terminal ammonium portion (B) (i.e., **2**,**4**,**6**,**7**) shared the loss of *m* 77.0842, *m* 87.0686, *m* 105.091, ascribable to the combined detachments of water, carbon monoxide and the ammonium group. A further neutral loss of *m* 131.0947 is probably due to a *trans*-acylation from the amide nitrogen to the oxygen on C2 and the consequent loss of alanine betaine as a neutral (Table S3, Supplementary Materials). The analogous neutral loss was not appreciated in the carunculines **1**,**3**,**5**,**8** mass spectra, as well as the combined losses of water, carbon monoxide and the ammonium group, confirming the structural difference between the two carunculine classes. Moreover, the higher number of isomers found for (B)-type carunculines agrees with the presence of a further stereogenic center at C1′ that enhances the number of possible stereoisomers.

### 2.2. Anatomical Distribution of Carunculines in H. carunculata

Given the support of the NMR data for the HPLC-ESI/HRMS identification of carunculines (**1**–**8**), the LC-HRMS data were considered as diagnostic to verify the anatomical distribution of these chemicals and their occurrence in other invertebrates.

**Figure 4:**
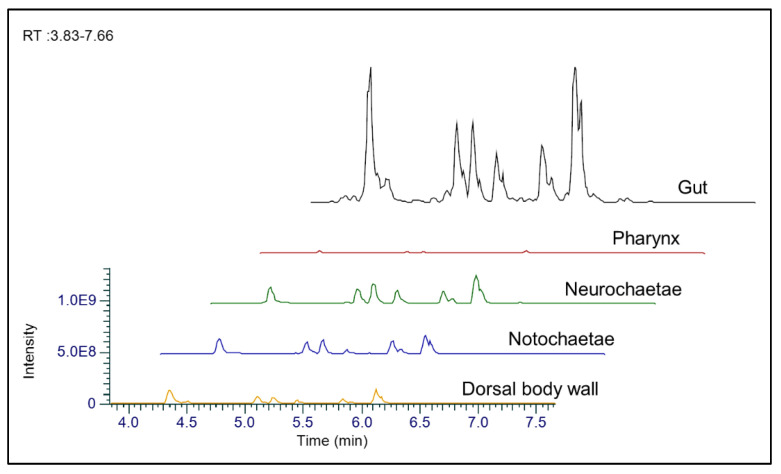
Anatomical distribution and relative abundance of carunculines in *H. carunculata* derived by HPLC-ESI/HRMS chromatograms and peak area counts.

The retention times and mass spectra in the *m*/*z* 283.2377 (**1**), *m*/*z* 271.2377 (**2**), *m*/*z* 285.2535 (**3**), *m*/*z* 273.2535 (**4**), *m*/*z* 309.2535 (**5**), *m*/*z* 297.2535 (**6**), *m*/*z* 299.2693 (**7**) and *m*/*z* 311.2693 (**8**) M^+^ ion chromatograms of *H. carunculata* body parts matched those identified from the fraction eluted by H_2_O+0.4% AcOH (Figure 4). Carunculines (**1**–**8**) and related isomers were consistently found within both the exposed tissues (i.e., dorsal body wall, noto- and neurochaetae) and the digestive apparatus (i.e., pharynx and gut). However, comparing the peak area counts, the concentration of the compounds seemed to vary considerably among the body parts. The peak area values reached their maxima in the gut in the case of carunculines **1**,**2**,**4**,**6**,**7**,**8**, followed by those detected in the noto- and neurochaetae. The dorsal body wall showed lower relative concentrations than the gut and the noto-/neurochaetae, but they were always higher than in the pharynx (Table S4, Supplementary Materials).

The relative abundance of the isomers varied a little among the body parts. For instance, i1 was the most abundant in all the tissues for carunculines **1**,**2**,**4**,**6**. In the case of carunculine **3**, i1 reached the highest peak area in the dorsal body wall, while it was exceeded by i2 in the noto-/neurochaetae and in the digestive tract (i.e., both gut and pharynx). For carunculine **7**, i1 was the greatest in the notochaetae, while i3 was the most abundant in all the other body parts (Table S4, Supplementary Materials).

### 2.3. Preliminary Screening for the Occurrence of Carunculines in Other Marine Invertebrates

The retention times and mass spectra in the ion chromatograms of extracts from *A. viridis*, *Perinereis* sp., *S. spallanzanii* and *Eisenia* sp., and *Sipunculus* sp. matched those corresponding with carunculines (**1**–**8**) detected in *H. carunculata* (Table S5, Supplementary Materials). Minor variations in RT were found in *Eisenia* sp., whose elution was characterized by a constant delay of about 0.20 s compared with the other invertebrates (Table S5, Supplementary Materials). Carunculines (as well as neocomplanines) were not detected in the samples of *B. brandaris*, *M. sabatieri* and *P. lividus*, as confirmed by semi-quantitative analyses of the peak area counts.

Comparing the peak area values, the concentration of carunculines (**1**–**8**) varied considerably among the invertebrate taxa. *H. carunculata* always had the highest concentrations, followed by *Sipunculus* sp., while *S. spallanzanii* and *Eisenia* sp. had much lower of peak area values, followed by *A. viridis,* where the lowest values were found (Table S5, Supplementary Materials).

Some differences in the distribution pattern of isomers were recorded. The most abundant in all the invertebrates was i1 for carunculines **2**,**6**, while i3 was consistently the highest in carunculine **7**. In the cases of carunculines **1**,**4**, i1 reached the highest peak area in most of the samples, including *A. viridis*, *Perinereis* sp., *S. spallanzanii*, *Eisenia* sp. and *H. carunculata* (where the area of i1 was almost the same as i2) (Table S5, Supplementary Materials).

## 3. Discussion

Extensive reviews have dealt with natural products from specific marine invertebrate groups, such as nudibranchs [30], cnidarians [5] and sponges [31]. However, the characterization of MNPs in marine annelids and insights into their bioprospecting potential are in the early stages. Attention has repeatedly been drawn to the importance of clarifying the venomous nature of amphinomid fireworms such as *Paramphinome jeffreysii*, *E. complanata* and *H. carunculata* [32]. Several toxins have been identified in bloodworms and amphinomids through transcriptomic analyses, revealing complex cocktails of putative toxin precursor transcripts [15,32]. However, complanine is the only strong skin-irritating non-proteinaceous molecule that has been isolated from an amphinomid so far [20].

For the first time, this study revealed a variety of novel natural compounds in *H. carunculata*. Thorough isolation work testing different extraction methods and chromatographic conditions was conducted, providing a total of at least eight new amino alcohols. These findings have exceeded expectations: *H. carunculata* displays a complex mixture of quaternary ammonium compounds with two different terminal structures: the former resembling glycine betaine (A) with Cα incorporated in a cyclopropane ring, and the latter derived from alanine betaine (B) (Figure 1b). Betaines are highly water-soluble compounds found in high quantities within marine invertebrates (such as mollusks, crustaceans and vestimentiferans), marine shallow-living osmoregulators, micro-organisms and plants [33,34]. They are usually known to be organic osmolytes involved in the protection of cells from osmotic stress, elevated temperature and unfavorable salt levels [35].

The vicinal amino alcohol motif is found in fatty acid derived sphingoid bases, which generate sphingolipids. Marine organisms represent a rich source of sphingolipids that lack the hydroxy group at C1 [36]. The structures of carunculines (**1**–**8**) are comparable to those detected in tunicates, such as obscuraminols from *Pseudodistoma obscurum* [23], crucigasterins from *Pseudodistoma crucigaster* [24], and clavaminols from *Clavelina phlegraea* [25,26]. In addition, alkyl amino alcohols and xestoaminols were isolated from the marine sponges *Haliclona* sp. and *Xestospongia* sp. [27,28], and spisulosine was found in the clam *Spisula polynima* [29]. As hypothesized for complanine, the similarity of these structures may suggest a close relationship in their biosynthetic pathways [37].

On HPLC-ESI/HRMS analysis, the carunculines were extremely hard to fragment, even testing different collision energies. The overall stability of these compounds was strongly supported further by NMR analyses on a sample left in D_2_O for more than one year, which showed no alteration in its ^1^H NMR spectrum [38].

The extraction of carunculines from both whole fireworm individuals and dissected body parts enabled us to determine whether these chemicals accumulate preferentially at specific sites. Compounds **1**–**8** were found in all fireworm body parts, with relative high concentrations in terms of peak areas in the gut and in the noto-/neurochaetae, which is involved in predator-prey interactions [17,19,39]. When disturbed, the chaetae become flared, creating a barrier that shields the entire fireworm body from contact, harms prey and triggers avoidance behavior in predators, thus protecting vital organs and avoiding serious damage [17,18]. In this way, the notochaetae could be deployed against enemies once easily released. Similar strategies occur in nudibranch mollusks, where defensive metabolites are stored in mantle dermal formations. These structures are accessible to predators and deliver highly distasteful compounds when broken, protecting organs that are crucial for the survival of the individual [40,41]. In the case of *H. carunculata*, these findings are in line with the low presence of carunculines in the dorsal body wall, which is protected by the flared tufts of chaetae and palatable, thus supporting the venomous nature of these chemicals that are not effective if ingested [18,19,39].

The significant amount found in the gut suggests an important role in the biosynthetic pathway of these chemicals. It appears that *de novo* synthesis of carunculine precursors may occur in the digestive apparatus starting from amino acids, like betaine, from organic matter ingested or taken directly from the environment [42], with subsequent methylation of Cα and attachment of the sphingosine derivatives. Indeed, the specimens employed in this study were kept in the lab for several months and fed only with defrosted fish prior to chemical analysis. This supports the hypothesis that *H. carunculata* should be capable of de novo synthesis of defensive secondary metabolites through a continuous production, rather than deriving them directly from dietary sources. However, even the production by symbiotic microbes might be possible, as in the case of several MNPs [31].

Carunculines seem to disappear, moving phylogenetically far away from the amphinomid group. Compounds 1–8 were found in the phyla Annelida (*H. carunculata*, *Sipunculus* sp., *S. spallanzanii*, *Eisenia* sp., *Perinereis* sp.) and Cnidaria (*A. viridis*), and were absent in echinoderms (*P. lividus*), mollusks (*B. brandaris*) and tunicates (*M. sabatieri*). Without a doubt, the greatest amounts were detected in fireworms, followed by sipunculids (the sister taxon of amphinomids [43]) and polychaetes belonging to the subclasses Sedentaria (i.e., earthworms and sabellids), where concentrations were one order of magnitude higher than in Errantia. Indeed, in the Errantia representative species (*Perinereis* sp.) the presence of carunculines was always extremely low and similar to those recorded in sea anemones. It is known that earthworms are characterized by a diversity of betaine compounds, among which glycine betaine is highly abundant and widespread in different earthworm taxa [44]. This may suggest the presence of a specialized system, maybe to prevent physiological stresses due to different environmental moisture levels [44].

The pattern of relative concentrations detected in annelids is thus consistent with the phylogenetic relationships occurring among the investigated taxa. Indeed, although several morphological characters separate the sister groups sipunculids and amphinomids, the intensities of carunculine ions reported for the former were at least two orders of magnitude higher than those of *Perinereis* sp., and equal to or lower than those detected in the pharynx of *H. carunculata*. Thus, we may assume that carunculines could be a plesiomorphy of the phylum Annelida, whose expression in non-amphinomid taxa seems to be extremely scarce. However, it cannot be excluded that these chemicals might also be present in other protostomes.

Calcareous chaetae have been considered a morphological apomorphy of amphinomids, together with nuchal organs on a sensory caruncle and the ventral eversible muscular pharynx [45,46]. The information provided in this study strongly support that the fragile, needle-like harpoon chaetae of the amphinomids constitute the crucial trigger giving rise to the effectiveness of venomous compounds. It is possible that the successful evolution of these chemicals in this lineage could have been pursued under positive Darwinian selection as a predatory adaptation, even with offensive roles [17,18]. Then, these chemicals may have encountered negative selection in Pleistoannelida taxa (i.e., Sedentaria and Errantia), the sister group of Sipuncula and Amphinomidae, which evolved alternative means of protection. For instance, many serpulids retract into strong tubes for refuge and their exposed body parts are unpalatable, while other polychaete species burrow deeply into the sediment [47].

Cnidaria are one of the most ancient animal phyla. A significant part of their ecological success can be attributed to a wide array of toxins and other bioactive molecules employed for defense and prey capture, which are stored and delivered by stinging cells, called “nematocysts” [48,49]. The venom discharged by the nematocysts appears to contain a variety of both proteinaceous and non-proteinaceous substances, including small molecules like quaternary ammonium compounds and betaines [50]. Also for the amphinomids, among the symptoms reported after urticating stings are neurotoxic effects and edema. Several sea anemone toxins have been well-characterized, while little is known about the small nonpeptidic molecules [51]. Thus, it should not be surprising that carunculines may also occur in this phylum.

Laboratory experiments and field observations have proven that fireworms are generalist predators of several Mediterranean invertebrate taxa, including cnidarians (anemones and corals), mollusks (nudibranchs, chitons), colonial ascidians and echinoderms (sea stars and sea urchins) [17,52]. Therefore, the occurrence of compounds one to eight in sea anemones and their pattern of distribution in *H. carunculata* may suggest the storage of chemicals directly from dietary prey, or biotransformation processes leading to more effective metabolites, as in the case of nudibranchs [41]. However, none of the compounds traced in fireworms have been detected in the other marine invertebrates they consume and from which toxins may possibly be retrieved. Furthermore, at equal sample concentrations, the intensity values of carunculines in sea anemones were much lower than in *H. carunculata*.

Overall, the present results support this array of metabolites, made effective by injection trough the stinging chaetae, as the extremely efficient predator avoidance strategy of *H. carunculata*, pursuing the ecological success of the species. Sphingoid-base derived amino alcohols exhibit interesting antifungal, anti-settlement and potent antimicrobial activity against diverse bacterial strains. In addition, spisulosine, obscuraminols and clavaminols showed cytotoxicity on tumor cell lines [36]. By capturing fireworms with ad hoc devices [53], further studies to test the biological activity of the set of carunculines (**1**–**8**) are ongoing to assess their effects starting from cellular models, while immunoassays could trace their transport inside fireworms to shed light on their biosynthetic pathways. It is also noteworthy that compounds one to eight differ from complanine and neocomplanines isolated from *E. complanata*, collected at Okinawa island (Japan [20]). Deeper chemical analyses are warranted to infer if within species and geographic variation of natural products may occur, since the range of natural compounds identified in *H. carunculata* suggest that even in other annelid taxa there might still be a lot to discover.

## 4. Materials and Methods

### 4.1. Animal Collection

*H. carunculata* has an amphi-Atlantic distribution, and is spread over the Central and Southern Mediterranean Sea and Eastern Atlantic Ocean. The specimens used in this study were collected by SCUBA diving at depths between 9–15 m in Apulia (40°7′27.90″ N, 17°59′47.89″ E; Central Mediterranean Sea, Italy). Gastropods (*Bolinus brandaris*), ascidians (*Microcosmus sabatieri*), sea urchins (*Paracentrotus lividus*), cnidarians (*Anemonia viridis*) and sedentary polychaetes (*Sabella spallanzanii*) were sampled by scraping rocky shores from the harbor of La Spezia (44°6′17.74″ N, 9°49′50.50″ E; Ligurian Sea, Italy). These marine invertebrates were purposely collected away from areas where fireworms are found [54] in order to avoid any possible chemical contamination due to the occasional interaction between the organisms considered (for instance, accidental consumption of small, dead or injured *H. carunculata* specimens).

The animals were transported separately in thermal containers with oxygenated seawater to the lab in Modena (Italy), where they were housed in different tanks of an aquarium with a recirculating system under controlled conditions (temperature 24–25 °C; salinity 32–36; photoperiod regime: 16 h light/8 h dark; total volume: 600 L). The fireworms were maintained for several months and fed ad libitum with defrosted large-scale sand smelt (*Atherina boyeri*) every two weeks. The other marine invertebrates were used in experiments the day after their arrival in the lab.

Errant polychaetes (*Perinereis* sp.), sipunculids (*Sipunculus* sp.) and earthworms (*Eisenia* sp.) were tested as additional representative taxa of the phylum Annelida. They were purchased (Pianeta Pesca, Modena, Italy) and used on the same day.

### 4.2. Isolation of Natural Compounds from H. carunculata

The fireworms were homogenized in distilled water (using a volume approximately equal to that of the worms), and dried at 60 °C per about 36 h.

The dried material was extracted at room temperature with acetone until the acetone solution appeared clear, then the solvent was evaporated under vacuum with a rotary evaporator (Rotavapor R-210A Buchi, Cornaredo, Italy). The concentrated extract was partitioned twice using a separating funnel and two different phases were tested: MeOH/H_2_O (9:1) against hexane; H_2_O/MeOH (9:1) against hexane first, and then EtOAc. The aqueous fractions were evaporated to obtain oily and crystallized residues, respectively.

Preliminary checks using HPLC-ESI/HRMS analysis confirmed that there were no carunculines present in the organic layers. The total aqueous extracts were chromatographed on a column packed with silica gel. A series of elution gradients were tested starting from [18], and trying both organo-halogenated solvents (mixtures of CHCl_3_ and MeOH) and no (mixtures of EtOAc and MeOH) (details on the elution gradients tested are reported in Table S1, Supplementary Materials). The ones that proved to be most effective were: the organo-halogenated mixtures (1) CHCl_3_, (2) CHCl_3_-MeOH (2:1), (3) CHCl_3_-MeOH-H_2_O-AcOH (10:5:1:0.06), (4) MeOH and (1) CHCl_3_, (2) CHCl_3_-MeOH (2:1), (3) CHCl_3_-MeOH (1:1), (4) CHCl_3_-MeOH (1:2), (5) MeOH; and the non-organo-halogenated mixture (1) EtOAc-MeOH (3:2), (2) EtOAc-MeOH (2:3), (3) EtOAc-MeOH (1:4), (4) MeOH, (5) H_2_O, (6) H_2_O + 0.4% AcOH.

Extensive chemical analyses had been conducted previously to ascertain the presence of any compounds (ions) that may be ascribable to complanine in *H. carunculata* (see [18] as an example). Notably, no spectrometric analyses were available on complanine in the literature to the best of our knowledge. Furthermore, considering the fact that it was impossible to retrieve standards, a proposal for the identification of chemicals from *H. carunculata* was achieved using HPLC-ESI/HRMS and NMR (600 MHz) analyses. The accurate *m*/*z* and molecular formula of the analytes identified were compared with those of complanine (C_18_H_35_N_2_O_2_^+^, monoisotopic mass 311.2693 [18,55]).

All the reagents used where purchased from Merck Life Science (Milano, Italy).

### 4.3. General Description of Analytical Techniques

The analyses for compound detection and identification were carried out using a high-pressure liquid chromatograph coupled to a high-resolution mass spectrometer via an electrospray ion source (HPLC-ESI/HRMS). The HPLC consisted of an UltiMate 3000 HPLC system equipped with anHPG 3400RS binary pump, an ISO 3100SD isocratic pump, a TCC 3000RS thermostatted column compartment, and a WPS 3000RS well plate sampler. The HRMS was a Q Exactive™ Hybrid Quadrupole-Orbitrap™ Mass Spectrometer equipped with a Heated Electrospray Ionization Source HESI-II probe (Thermo Fisher Scientific, Waltham, MA, USA).

Chromatographic separation was accomplished by using a 100 × 2.1 mm ID 3.5 μm ps Zorbax Reversed-Phase Extended C18 Column (Agilent Technologies, Santa Clara, CA, USA) with a flow rate of 0.4 mL/min and a linear gradient of solvent A (H_2_O + 0.1% formic acid) and solvent B (acetonitrile + 0.1% formic acid). Following a 1 μL injection, the chromatographic run started at 10% eluent B, which was kept for 0.5 min then raised to 85% B in 15.5 min and to 90% B in 0.1 min; 90% B was kept until 20 min, then the starting 10% B condition was restored in 0.1 min and the system was conditioned for a further 10 min pending successive injection.

High-resolution accurate-mass spectra were acquired in positive electrospray ionization mode (ESI+) using two alternating acquisition functions: Full MS and Parallel Reaction Monitoring (PRM). The Full MS scan acquisition events recorded centroid mass spectra with a resolving power of 35,000 full width at half-maximum (FWHM) at *m*/*z* 200 in the *m*/*z* range from 150 to 2000. These scans were performed using an Automatic Gain Control (AGC) target of 1 million charges (1 × 10^6^) with a maximum injection time (IT) of 123 ms. Mass-spectrometry based identification of carunculines followed a two-step approach. Given the high resolution and high mass accuracy (35,000 FWHM at *m*/*z* 200 and inaccuracy < 3 ppm, respectively) of Full MS and MS/MS spectra, the exact molecular formulae were determined computationally for the ions revealed [56]. In the case of the MS spectra containing “precursor ions”, this enabled us to hypothesize the chemical formulae of the molecules which generated them under ESI+ conditions, constituting the first step for the characterization of the carunculines. As a second step, when full-scan data had been obtained and the exact masses and RT for precursor ions of interest were known, it was possible to generate the inclusion list for the PRM acquisitions of MS/MS spectra.

The PRM acquisitions were performed to acquire an MS/MS scan (fragmentation spectra) of the carunculines and were triggered by a specific time-scheduled precursor inclusion list built on the basis of preliminary analyses. The list was composed of the monoisotopic ions at *m*/*z* 283.23770 (RT range 3.70–4.80 min), *m*/*z* 271.23770 (3.80–4.70 min), *m*/*z* 285.25350 (4.50–5.50 min), *m*/*z* 273.25350 (4.50–5.60 min), *m*/*z* 309.25350 (4.80–5.80 min), *m*/*z* 297.25350 (4.80–5.80 min), *m*/*z* 299.26930 (5.30–6.20 min) and *m*/*z* 311.26930 (5.60–7.00 min).

Xcalibur 2.0 (Thermo Fisher Scientific, Waltham, MA, USA) was used for the data acquisition, inclusion list building and data processing (FreeStyle 1.5 software, Thermo Fisher Scientific, Waltham, MA, USA). Extracted ion chromatograms (XICs) were generated using a 5 ppm mass window centered on the exact *m*/*z* of each analyte.

The exact mass and RT of the carunculines were used to perform retrospective analyses on full-scan data to evaluate the presence of these compounds, both in *H. carunculata* body parts and other marine invertebrate taxa [56]. The relative quantification of the carunculines across the different samples was performed using XICs of their relative precursor ions in the Full MS spectra. The carunculines were revealed by chromatographic peaks with specific *m*/*z*—RT coordinates. The peak area values are proportional to concentration and were used to address the relative expression of the carunculines in the different samples considered. Each peak area value was calculated using the computer data station with peaks automatic integration and manual verification using the QuanBrowser software (Thermo Fisher Scientific, Waltham, MA, USA) in the Xcalibur package. Peaks were identified by extracting the exact mass of the expected ion with a mass tolerance of 5 ppm from the mass range trace of the Full-MS data.

The complex mixture of carunculines was analyzed using NMR to achieve structure elucidation of compounds. The NMR spectra were acquired in D_2_O on a FT-NMR AVANCE III HD 600 MHz spectrometer (Bruker Biospin, Billerica, MA, USA) operating at 600.13 and 150.90 MHz for ^1^H and ^13^C, respectively, at 298 K equipped with a cryoprobe and pulse field gradients. ^1^H and ^13^C chemical shifts are referenced to the methyl signal of the acetate ion set at 1.904 and 24.0 ppm, respectively. 2D NMR experiments were carried out using standard pulse sequences (Bruker library): cosygpprqf (H,H-COSY), mlevphpr (H,H-TOCSY, 110 ms spin-lock time), hsqcedetgpsp.3 (H,C-HSQCed), noesygpphpr (H,H-NOESY, 450 ms mixing time), hmbcetgpl2nd (H,C-HMBC, 62.5 ms evolution time), hsqcetgp (H,N-HSQC, 25 ms evolution time) and hsqcdietgpsisp (H,C-HSQC-TOCSY, 110 ms spin-lock time).

### 4.4. Anatomical Distribution of Fireworm Toxins

Adult specimens of *H. carunculata* were dissected to look for the presence of carunculines in both overt body parts (the dorsal body wall), which are involved in predator-prey interactions (noto- and neurochaetae), and in the digestive apparatus (divided into pharynx and gut) (see [18] and Figures S10 and S11, Supplementary Materials, for a description of the dissection protocol). The same body parts obtained from different individuals were pooled, and each tissue type was investigated separately (see Table S6, Supplementary Materials, for the weights of the freeze-dried samples and acetone extracts).

Each tissue was homogenized with distilled water and freeze-dried. The freeze-dried material was extracted with acetone until the acetone solution appeared clear. The evaluation of the anatomical distribution of carunculines was assessed by screening with HPLC-ESI/HRMS, given that obtaining samples for NMR analysis would have required the killing of several *H. carunculata* specimens unnecessarily.

### 4.5. Occurrence of Carunculines in Other Marine Invertebrate Taxa

Whole individuals of *H. carunculata*, *A. viridis*, *Perinereis* sp., *Sipunculus* sp. and *Eisenia* sp. and soft tissues of *P. lividus*, *B. brandaris*, *S. spallanzanii* and *M. sabateri* were rinsed with marine water to remove accidental impurities whenever necessary, and then directly homogenized with distilled water. Individuals belonging to the same species were pooled and each taxon was investigated separately. Each homogenized sample was freeze-dried and extracted with acetone until the acetone solution appeared clear. Obtaining sufficient amounts of extract for structure elucidation using NMR analysis proved to be a difficult task (see Table S6, Supplementary Materials, for the weights of freeze-dried samples and acetone extracts). Therefore, as well as for the analysis of fireworm body parts, the occurrence of carunculines was verified with HPLC-ESI/HRMS using the data obtained from in toto specimens of *H. carunculata* as a reference.

## Figures and Tables

**Figure 2:**
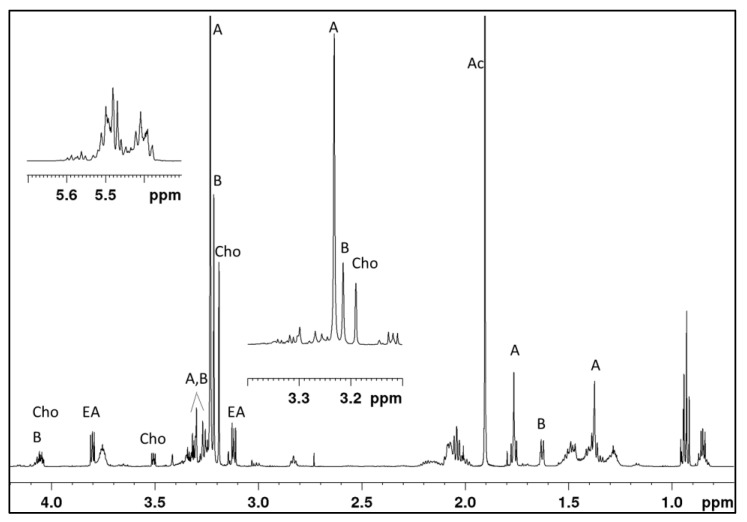
^1^H spectrum in D_2_O of the fraction eluted by H_2_O + 0.4% AcOH of *H. carunculata* extract. Diagnostic signals of carunculines are marked with A and B. Cho = choline, EA = ethanolamine.

## Supplementary Materials

The following supporting information can be downloaded at: https://www.mdpi.com/article/10.3390/md20090585/s1, Figure S1 (a–p): extracted ion chromatograms of carunculines (**1**–**8**) (a,c,e,g,i,k,m,o) and related spectra (b,d,f,h,j,l,n,p), Table S1: different mixtures of solvents (mobile phases) tested for column chromatography on silica gel, Figure S2: Assignments of ^1^H, ^13^C and ^15^N (red) NMR signals in terminal ammonium groups (A and B) and alkyl chains (I–VI) derived by NMR analysis, Figure S3: Enlarged regions of the H,C-HSQCed NMR spectrum, Figure S4: H,N-HSQC NMR spectrum of carunculines, Figure S5: H,H-COSY NMR spectrum of carunculines, Figure S6: Partial H,C-HMBC NMR spectrum of carunculines, Figure S7: H,H-TOCSY NMR spectrum of carunculines, Figure S8: H,H-NOESY NMR spectrum of carunculines, Figure S9: H,C-HSQC-TOCSY NMR spectrum of carunculines, Table S2: Relevant MS/MS fragments identified in carunculines (**1**,**3**,**5**,**8**) using HPLC-ESI/HRMS, Table S3: Relevant MS/MS fragments identified in carunculines (**2**,**4**,**6**,**7**) using HPLC-ESI/HRMS, Table S4. Carunculines (**1**–**8**) and their relative quantification in fireworm tissues based on peak area counts. The highest peak area value for each isomer was counted as 100% and the others were derived in proportion, Table S5. Carunculines (**1**–**8**) and their relative quantification in marine invertebrates based on peak area counts. The highest peak area value for each isomer was counted as 100% and the others were derived in proportion, Figure S10 (A–E): dissection of the pharynx, Figure S11 (A–H): dissection of the dorsal body wall and gut, Table S6 weight of the freeze-dried samples and acetone extracts obtained from the body parts of *H. carunculata* and from the marine invertebrate taxa examined.
