# Supplementary material for: Novel Natural Compounds and Their Anatomical Distribution in the Stinging Fireworm Hermodice carunculata (Annelida)
Source: Mar Drugs. 2022 Sep 19;20(9):585. doi: 10.3390/md20090585 (PMC9504318; doi:10.3390/md20090585)
Supplement: Supplementary file 1 [file marinedrugs-20-00585-s001.zip › marinedrugs-1888805-supplementary.pdf]

Supplementary material to

Novel Natural Compounds and Their Anatomical Distribution in the Stinging Fireworm *Hermodice carunculata* (Annelida)

Authors

Sara Righi [1,*], Luca Forti [2], Roberto Simonini [1], Valentina Ferrari [1], Daniela Prevedelli [1] and Adele Mucci [3]

[1] Department of Life Sciences, University of Modena and Reggio Emilia, Via Campi 213/D, 41125 Modena, Italy
[2] Department of Life Sciences, University of Modena and Reggio Emilia, Via Campi 103, 41125 Modena, Italy
[3] Department of Chemical and Geological Sciences, University of Modena and Reggio Emilia, Via Campi 103, 41125 Modena, Italy
* Correspondence: sara.righi@unimore.it

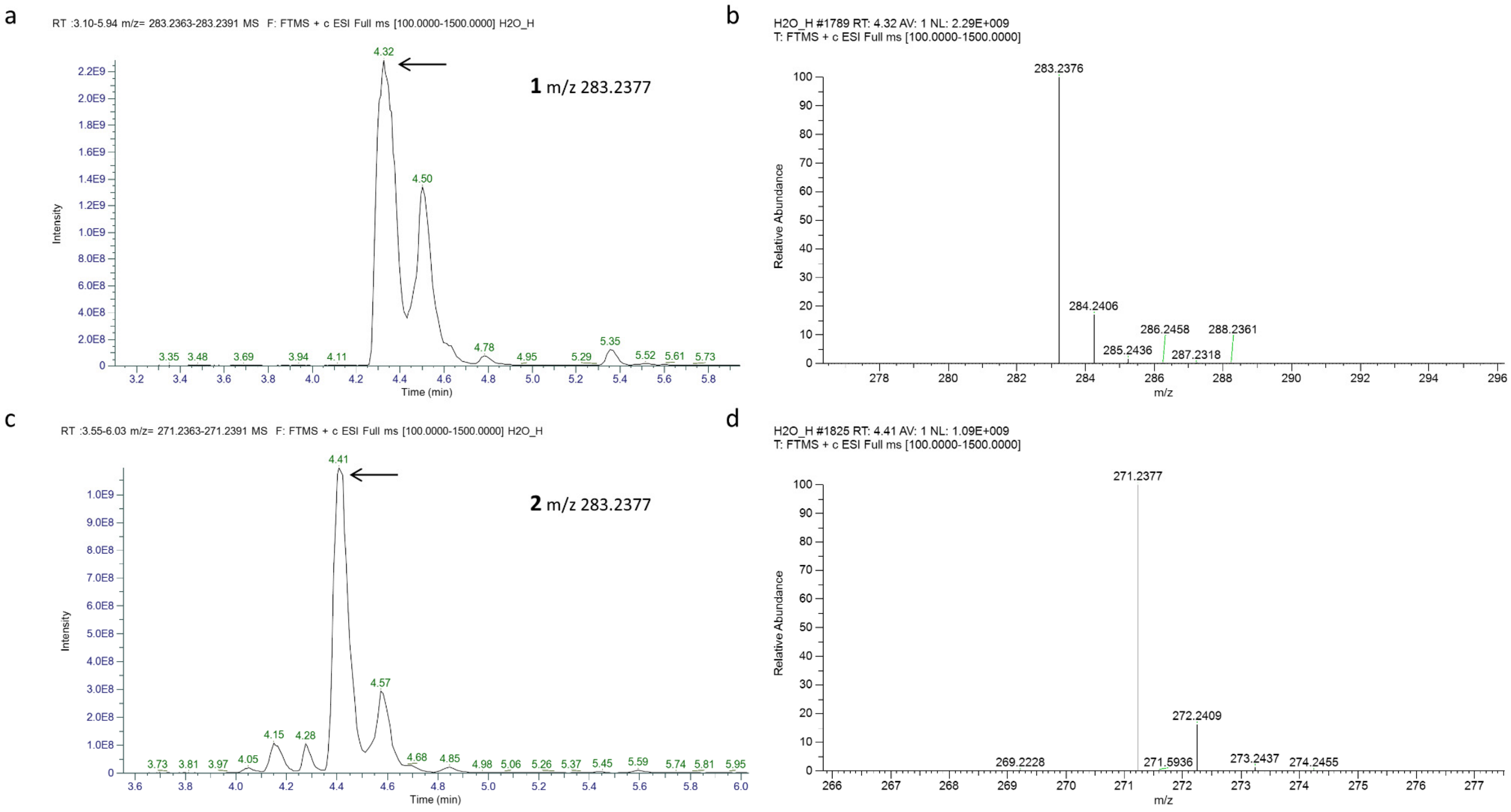

Figure S1 (a-l). Extracted ion chromatograms of carunculines (**1-8**) (a,c,e,g,i,k,m,o) and related spectra (b,d,f,h,j,l,n,p). When multiple isomers occurred, the black arrows indicate the peak to which the spectrum is referred.

e

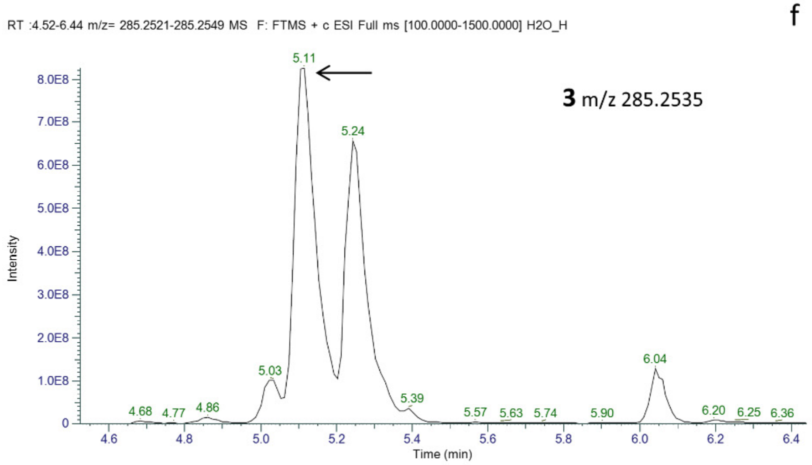

f

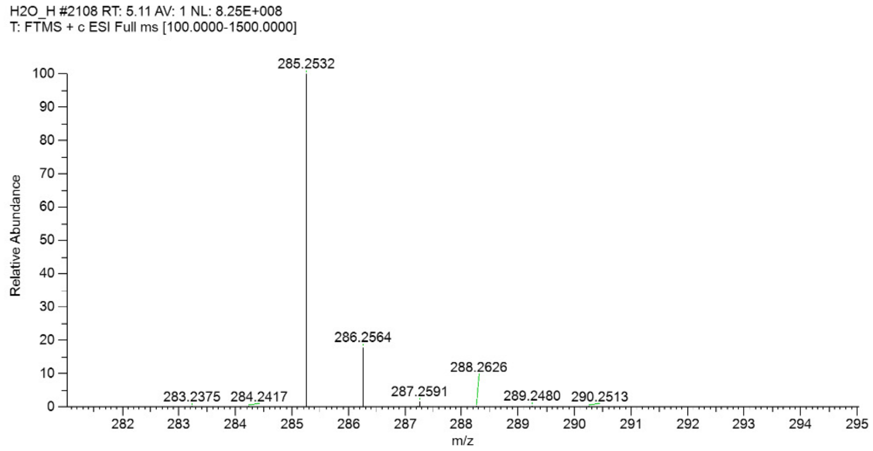

g

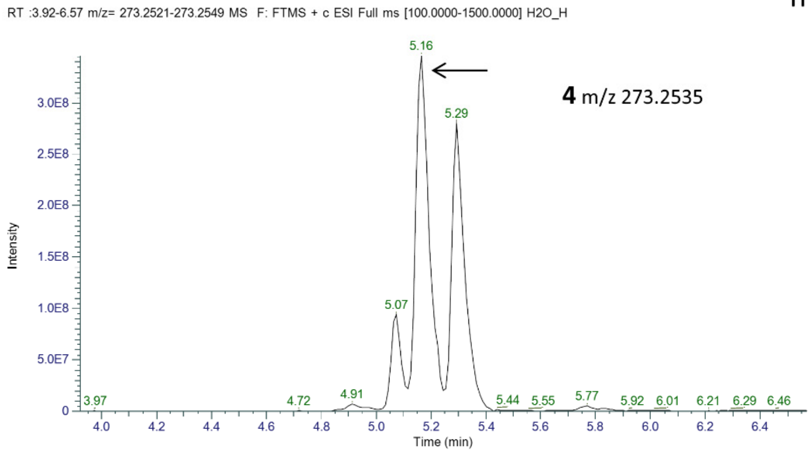

h

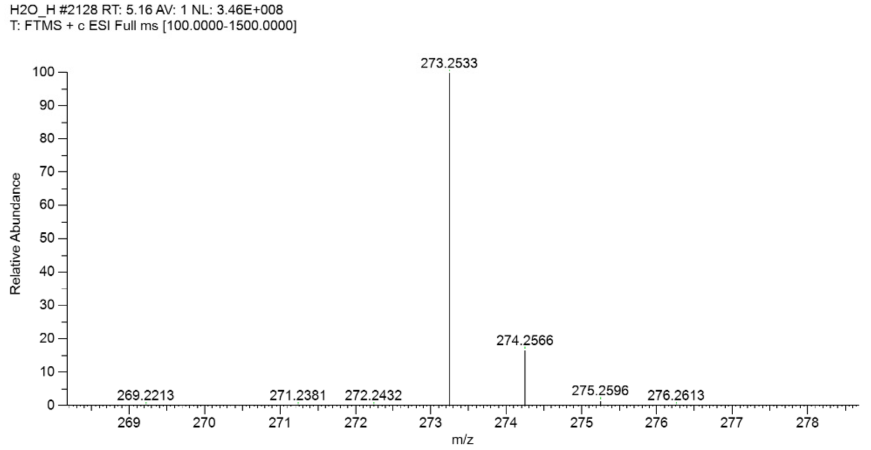

Figure S1 (a-l). Continue.

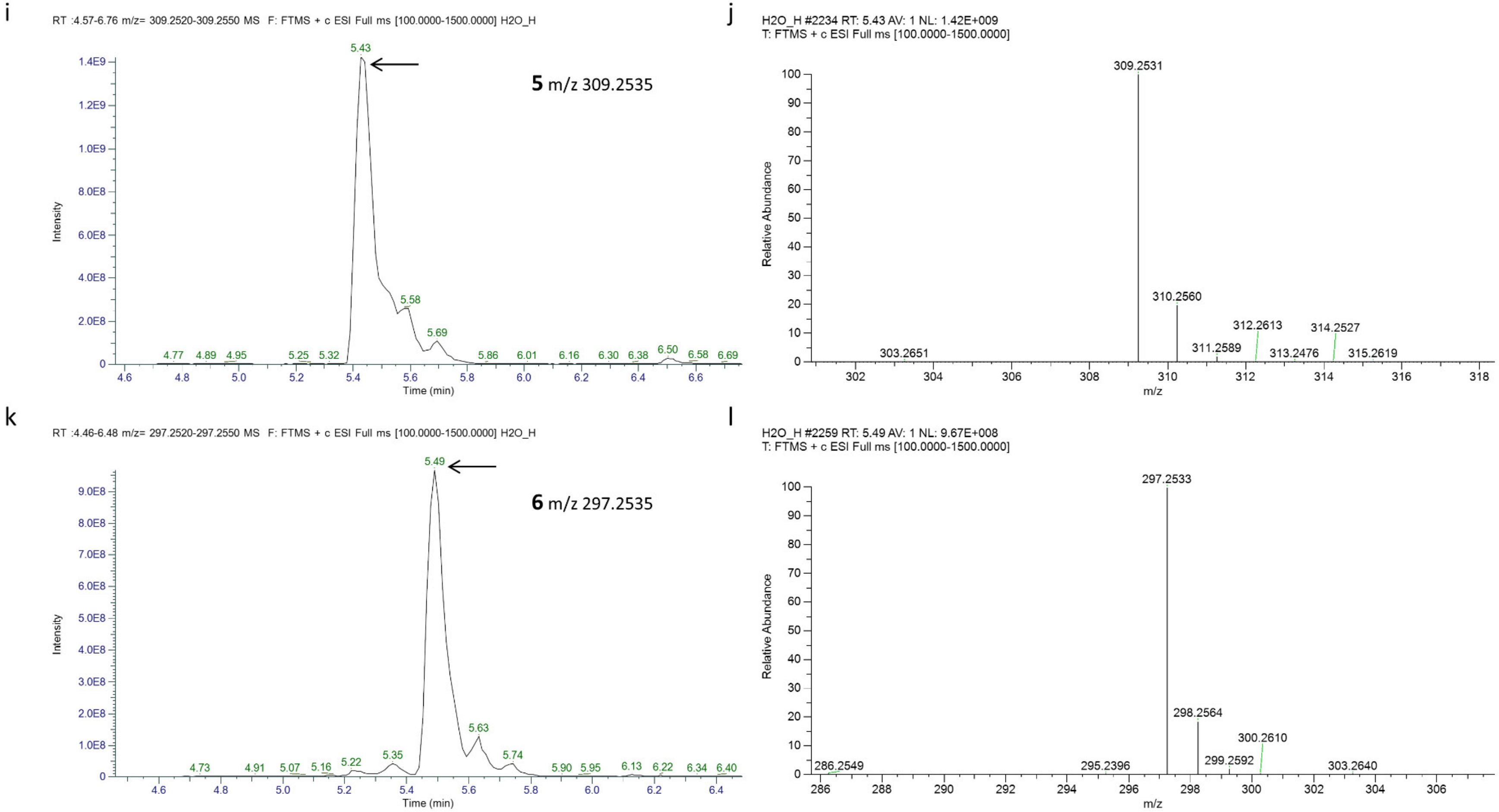

Figure S1 (a-l). Continue.

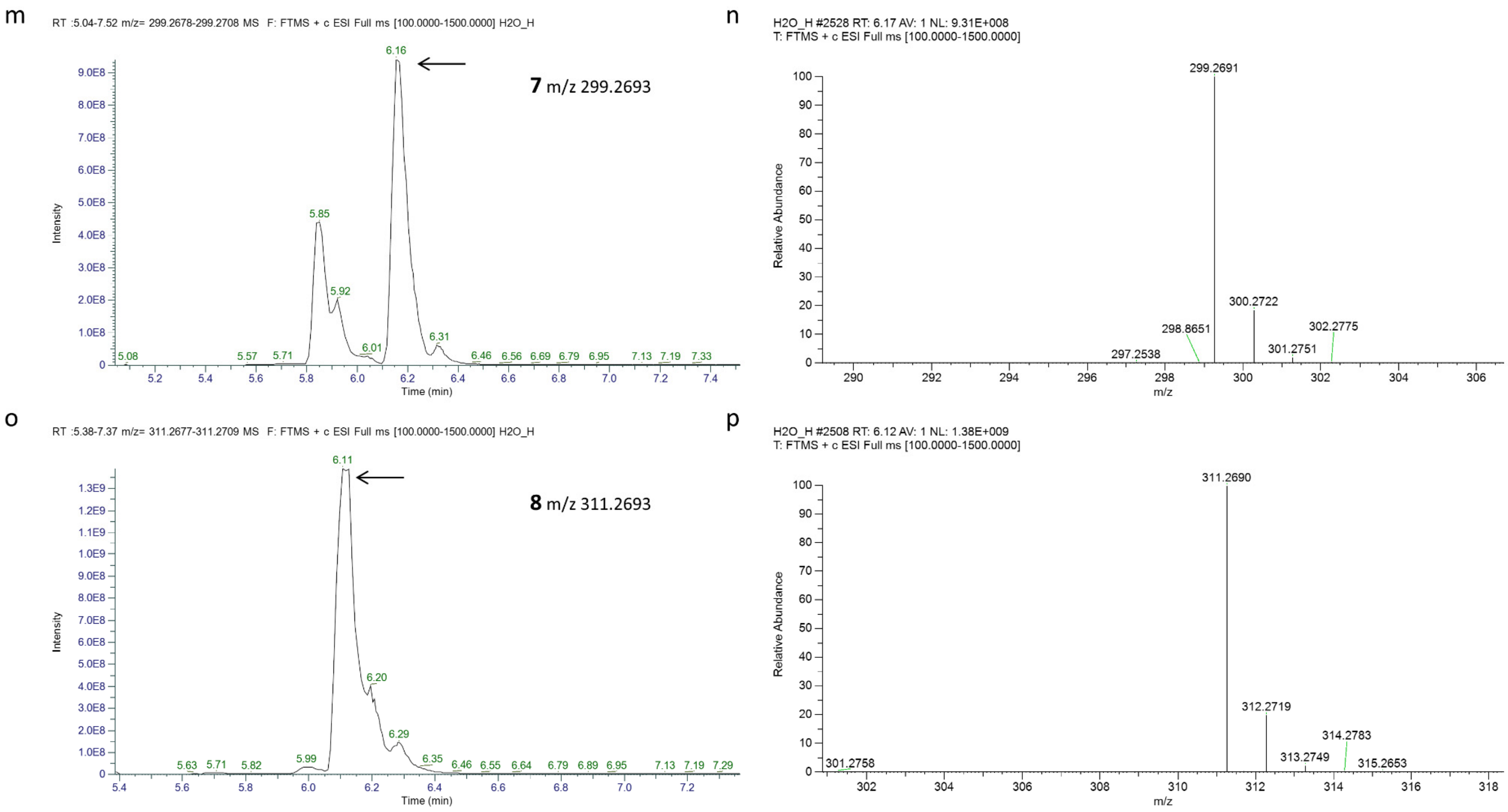

Figure S1 (a-l). Continue.

Table S1. Different mixtures of solvents (mobile phases) tested for column chromatography on silica gel. The weight of the extracts after partitioning and of singular fractions ("Fr") are reported. EtOAc = ethyl acetate; AcOH = acetic acid.

Test n.	Extract after partitioning (mg)	Mobile phase	Fr 1 (mg)	Fr 2 (mg)	Fr 3 (mg)	Fr 4 (mg)	Fr 5 (mg)	Fr 6 (mg)	Fr 7 (mg)
1A	2738.0	**1)** $CHCl_3$, **2)** $CHCl_3$-MeOH (2:1), **3)** $CHCl_3$-MeOH-H_2O-AcOH (10:5:1:0.06), **4)** MeOH	12.9	15.3	447.0	154.0	/	/	/
1B		**1)** $CHCl_3$-MeOH (3:1) **2)** $CHCl_3$-MeOH (2:1), **3)** $CHCl_3$-MeOH (1:1), **4)** $CHCl_3$-MeOH (1:2), **5)** MeOH	15.4	11.9	18.1	16.9	8.1	/	/
2	*	**1)** $CHCl_3$, **2)** $CHCl_3$-MeOH (2:1), **3)** $CHCl_3$-MeOH (1:1), **4)** $CHCl_3$-MeOH (1:2), **5)** MeOH	*	169.0	64.0	38.0	13.0	/	/
3	812.0	**1)** $CHCl_3$-MeOH (3:1), **2)** $CHCl_3$-MeOH (2:1), **3)** MeOH	266.0	243.0	79.0	/	/	/	/
4	291.1	**1)** CH_2Cl_2-MeOH (3:1), **2)** CH_2Cl_2-MeOH (2:1), **3)** CH_2Cl_2-MeOH (1:1), **4)** CH_2Cl_2-MeOH (1:2), **5)** MeOH, **6)** H_2O, **7)** H_2O+0.4% AcOH	38.1	22.6	17.2	29.1	14.7	19.0	19.2
5A	932.0	**1)** EtOAc-MeOH (4:1), **2)** EtOAc -MeOH (3:2), **3)** EtOAc -MeOH (2:3), **4)** EtOAc -MeOH (1:4), **5)** MeOH	61.4	41.9	33.6	14.4	13.0	/	/
5B		**1)** EtOAc -MeOH (3:2), **2)** EtOAc -MeOH (2:3), **3)** EtOAc-MeOH (1:4), **4)** MeOH, **5)** H_2O, **6)** H_2O+0.4% AcOH	274.2	73.8	52.4	6.6	6.6	43.9	/

* Not available due to accidental solvent bumping.

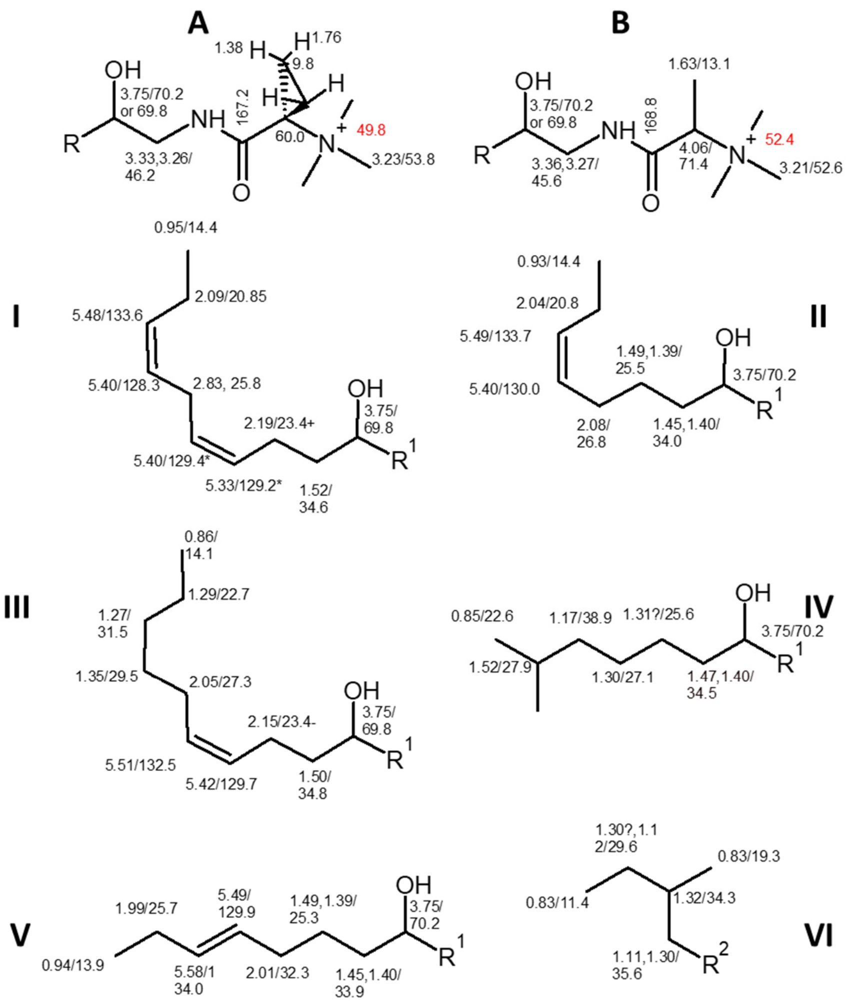

Figure S2. Assignments of ^{1}H, ^{13}C and ^{15}N (red) NMR signals in terminal ammonium groups (A and B) and alkyl chains (I-VI) derived by NMR analysis.

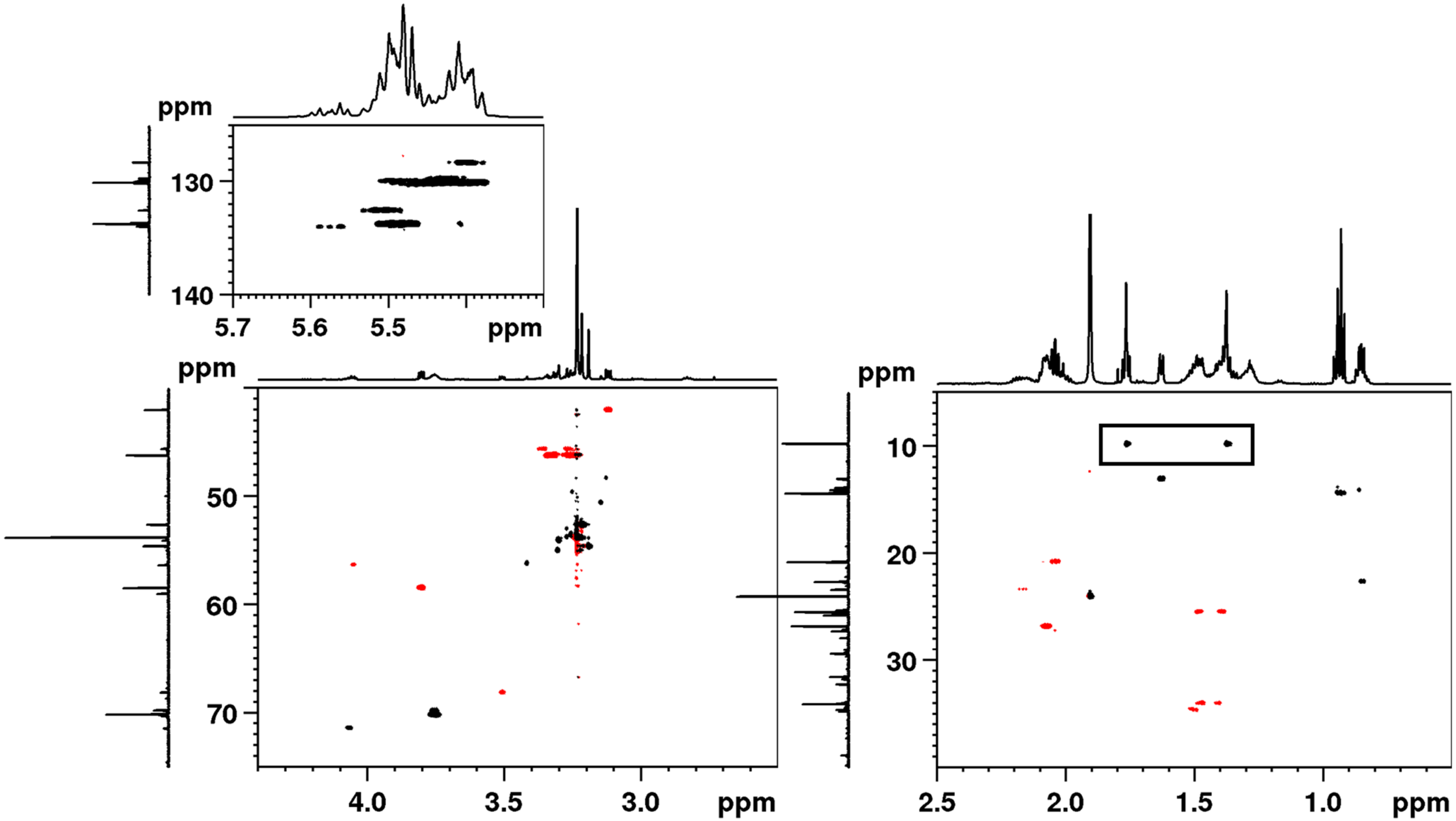

Figure S3. Enlarged regions of the H,C-HSQCed NMR spectrum. In the black rectangle, the correlation between the protons and the carbon of the cyclopropane ring, with the same sign of methyl and methyne correlations.

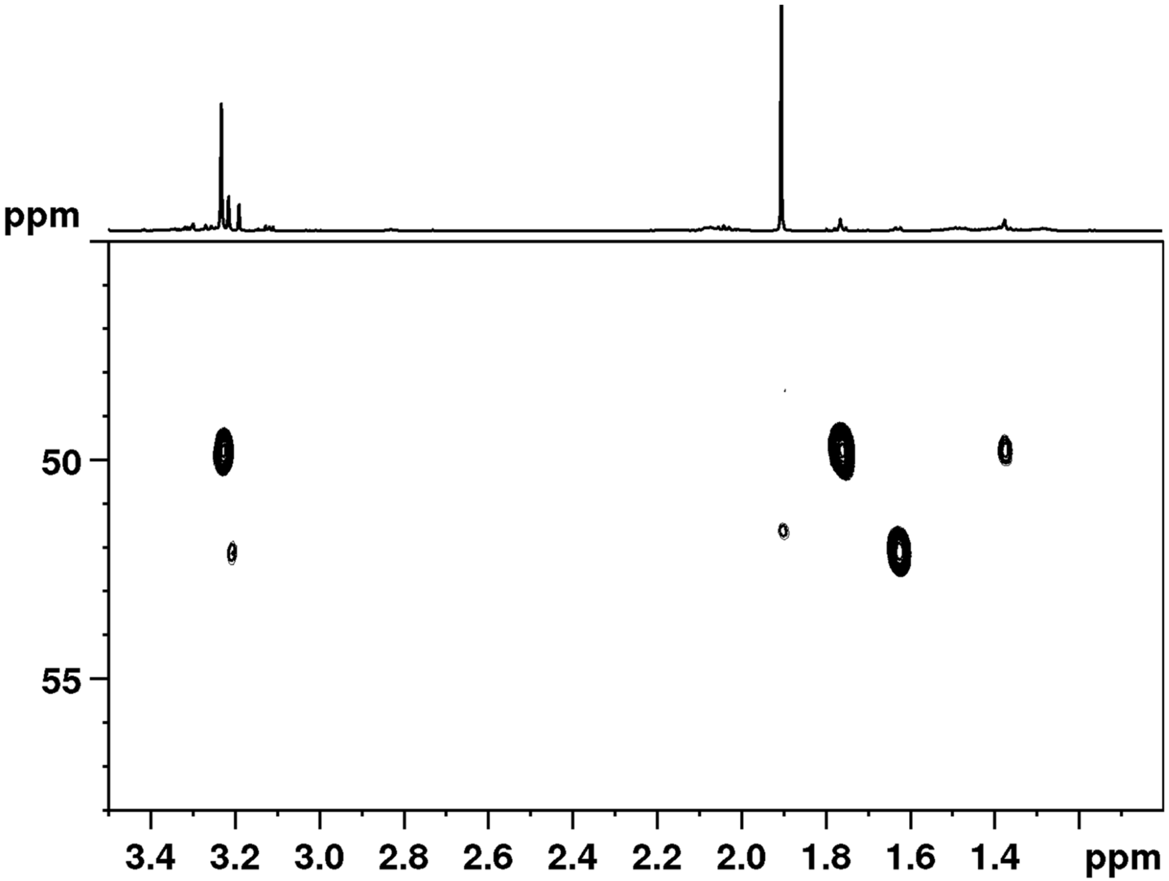

Figure S4. H,N-HSQC NMR spectrum of carunculines.

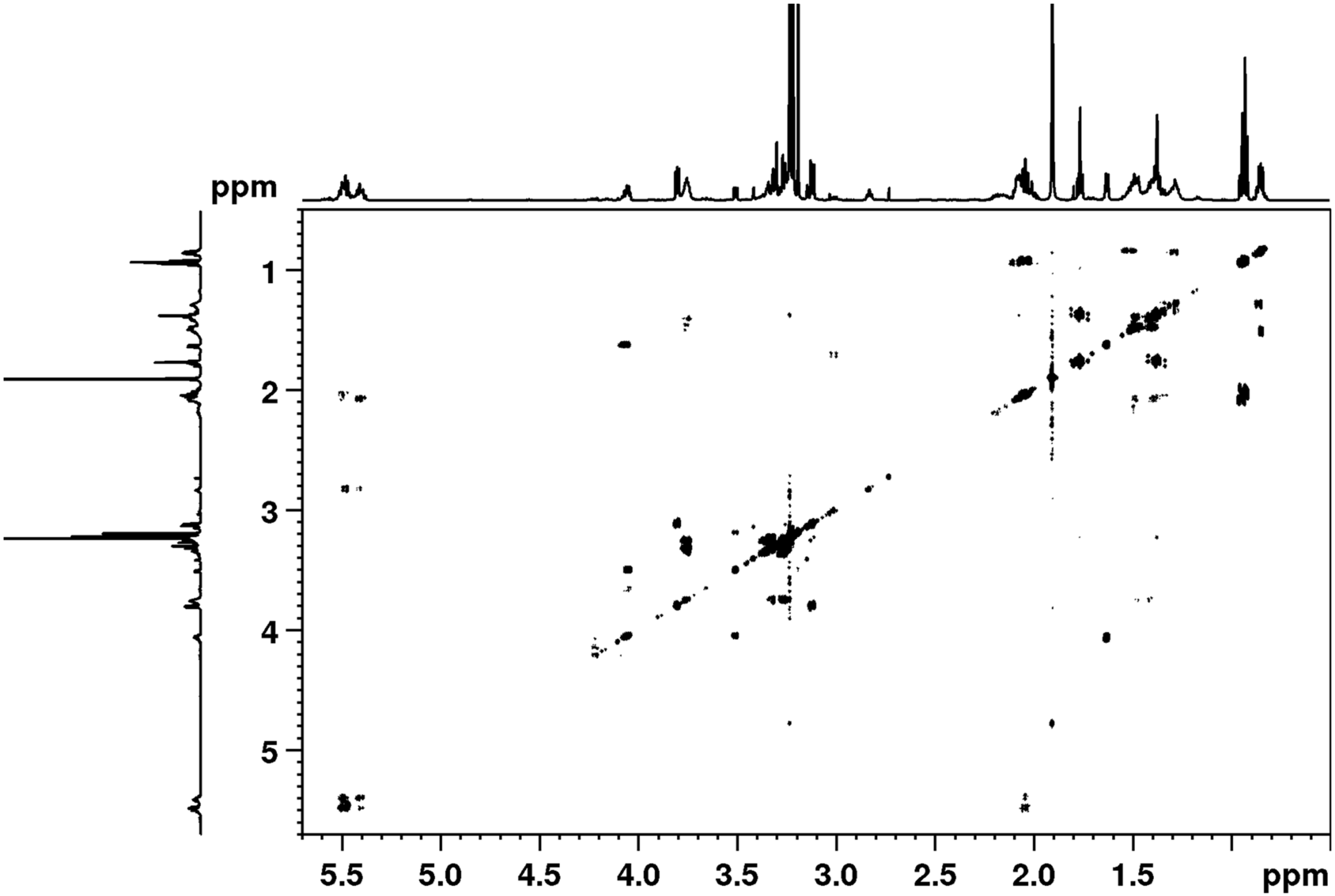

Figure S5. H,H-COSY NMR spectrum of carunculines.

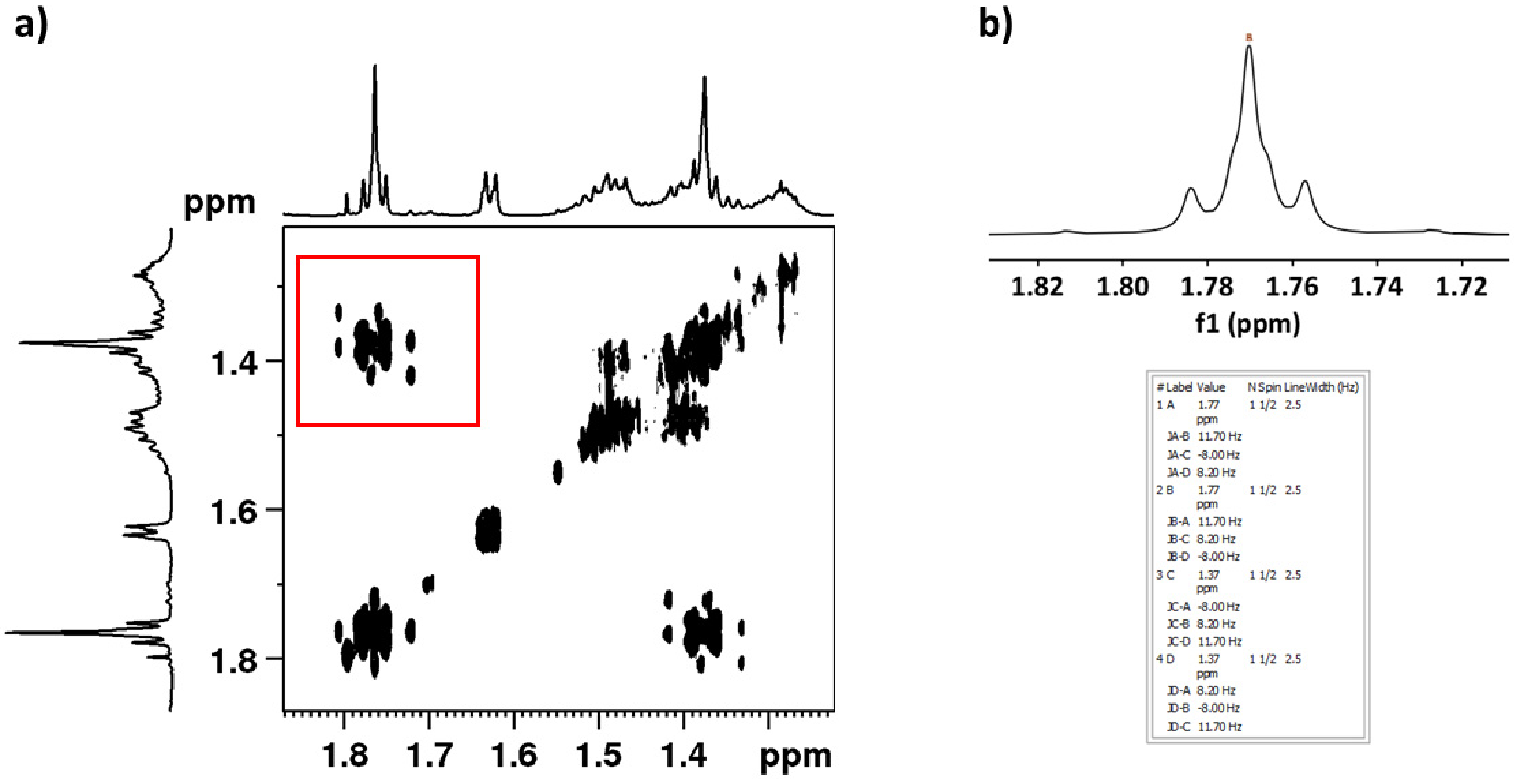

Figure S5 (continue). a) Enlarged region of the H,H-COSY NMR spectrum of carunculines. The red square shows the correlation between the AA′ and BB′ protons of the cyclopropane ring. b) spin-system simulation showing the shape of one of the two multiplets of the AA′BB′ system (MestReNova v 14.1.2-25024).

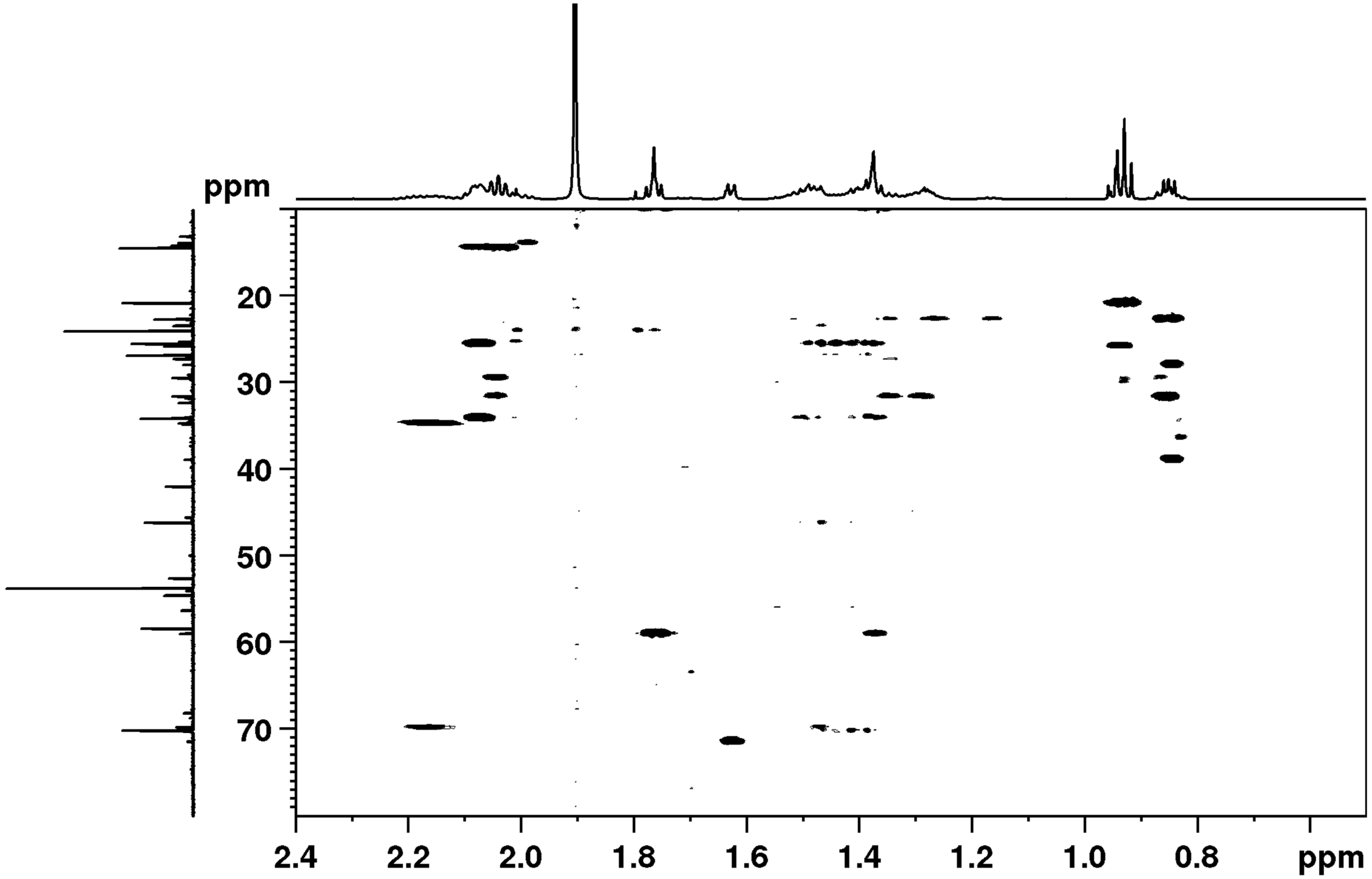

Figure S6. Partial H,C-HMBC NMR spectrum of carunculines.

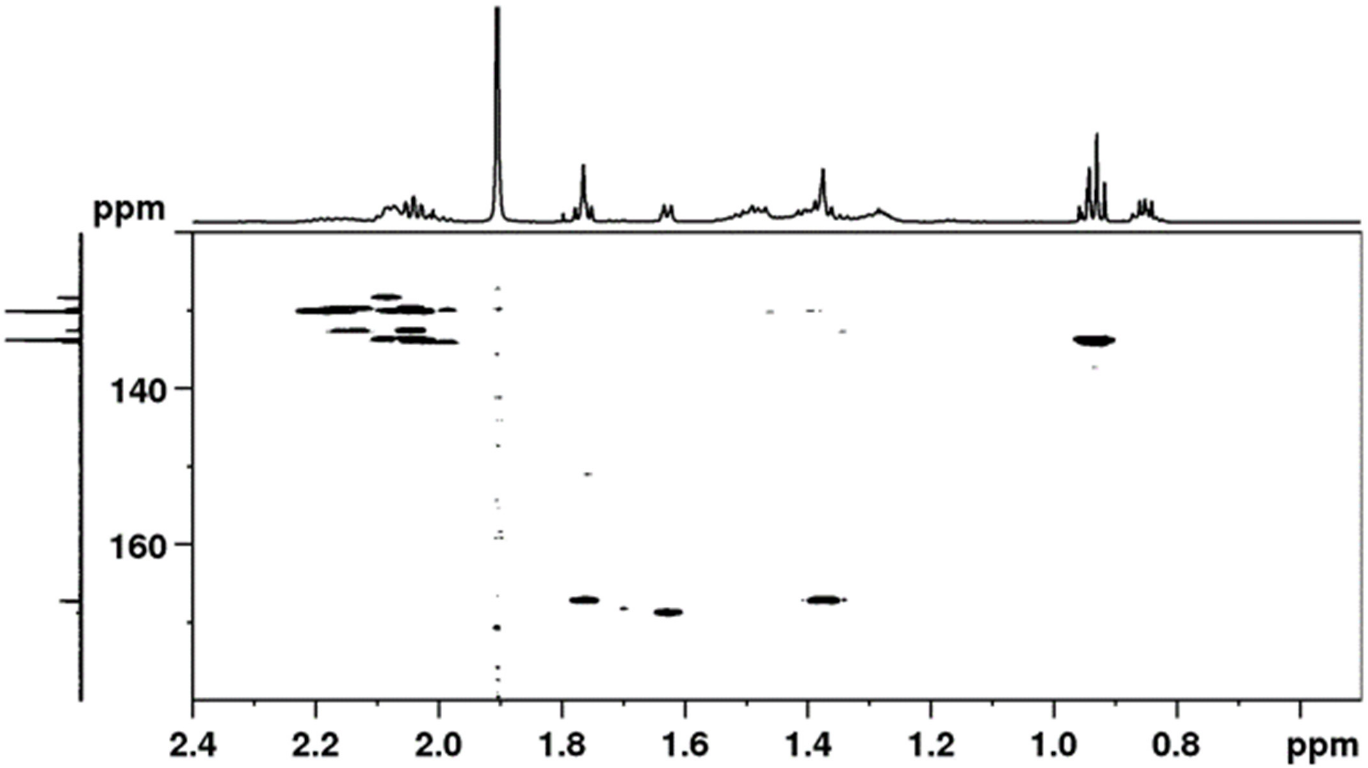

Figure S6 (continue). Partial H,C-HMBC NMR spectrum of carunculines.

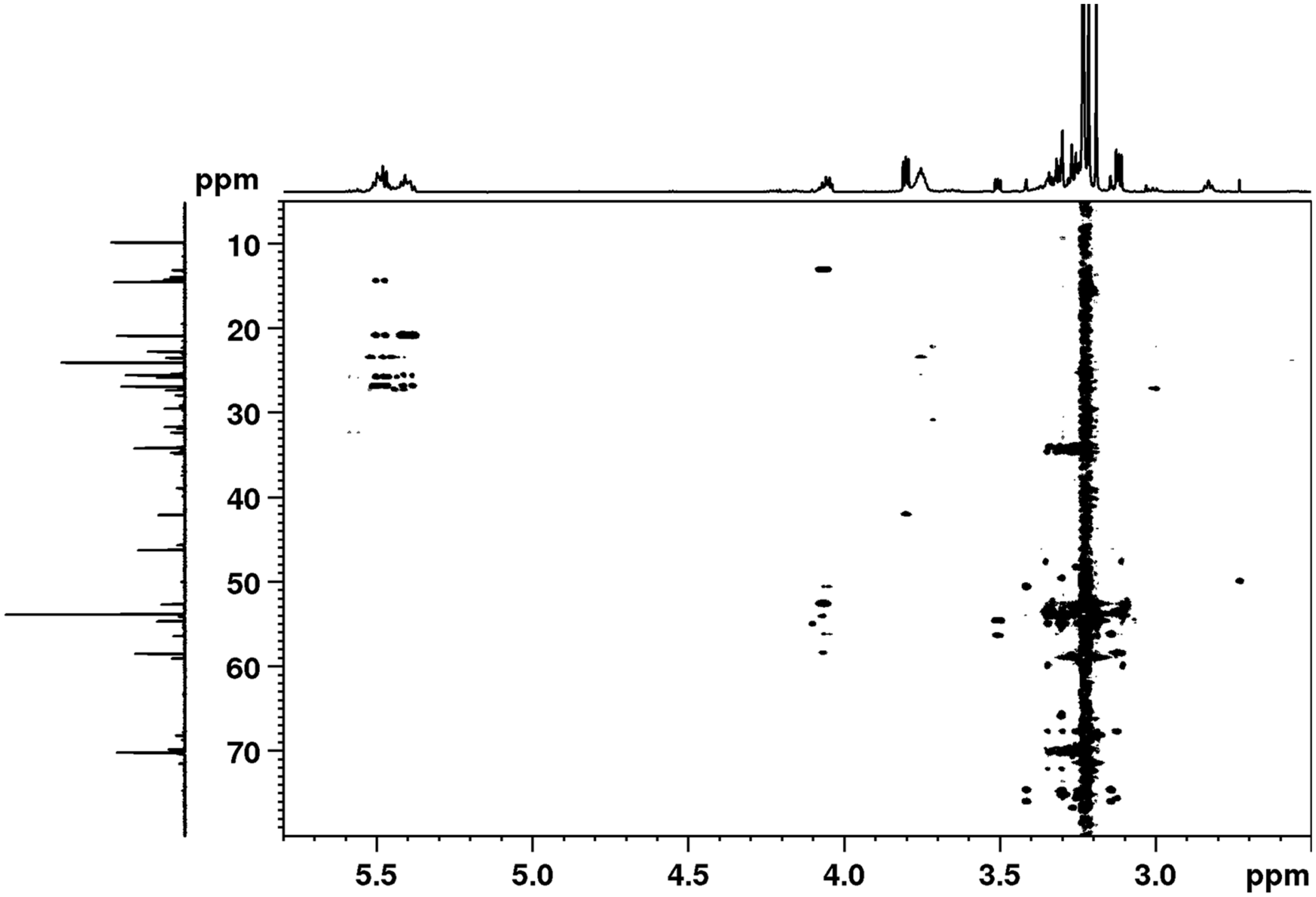

Figure S6 (continue). Partial H,C-HMBC NMR spectrum of carunculines.

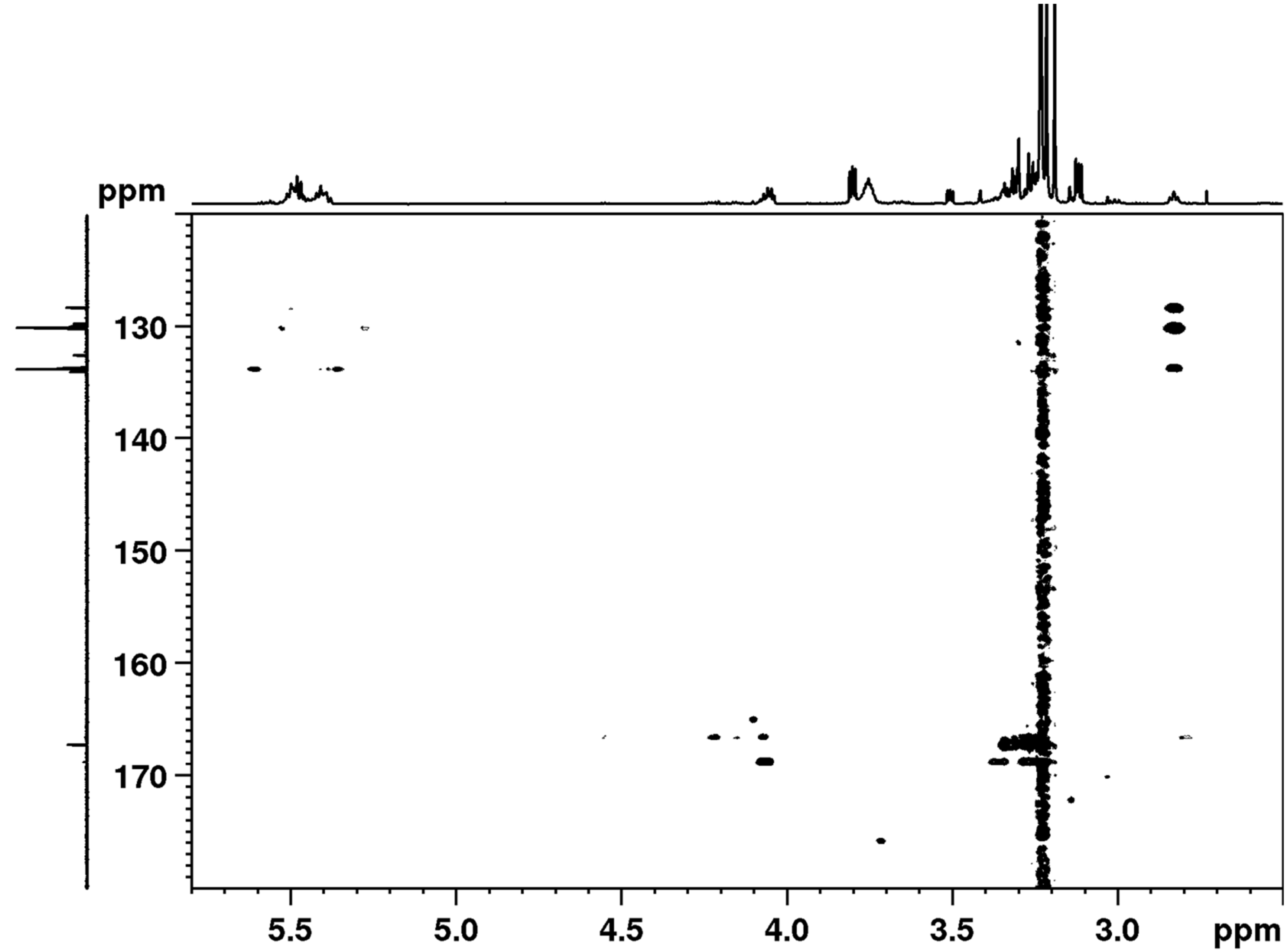

Figure S6 (continue). Partial H,C-HMBC NMR spectrum of carunculines.

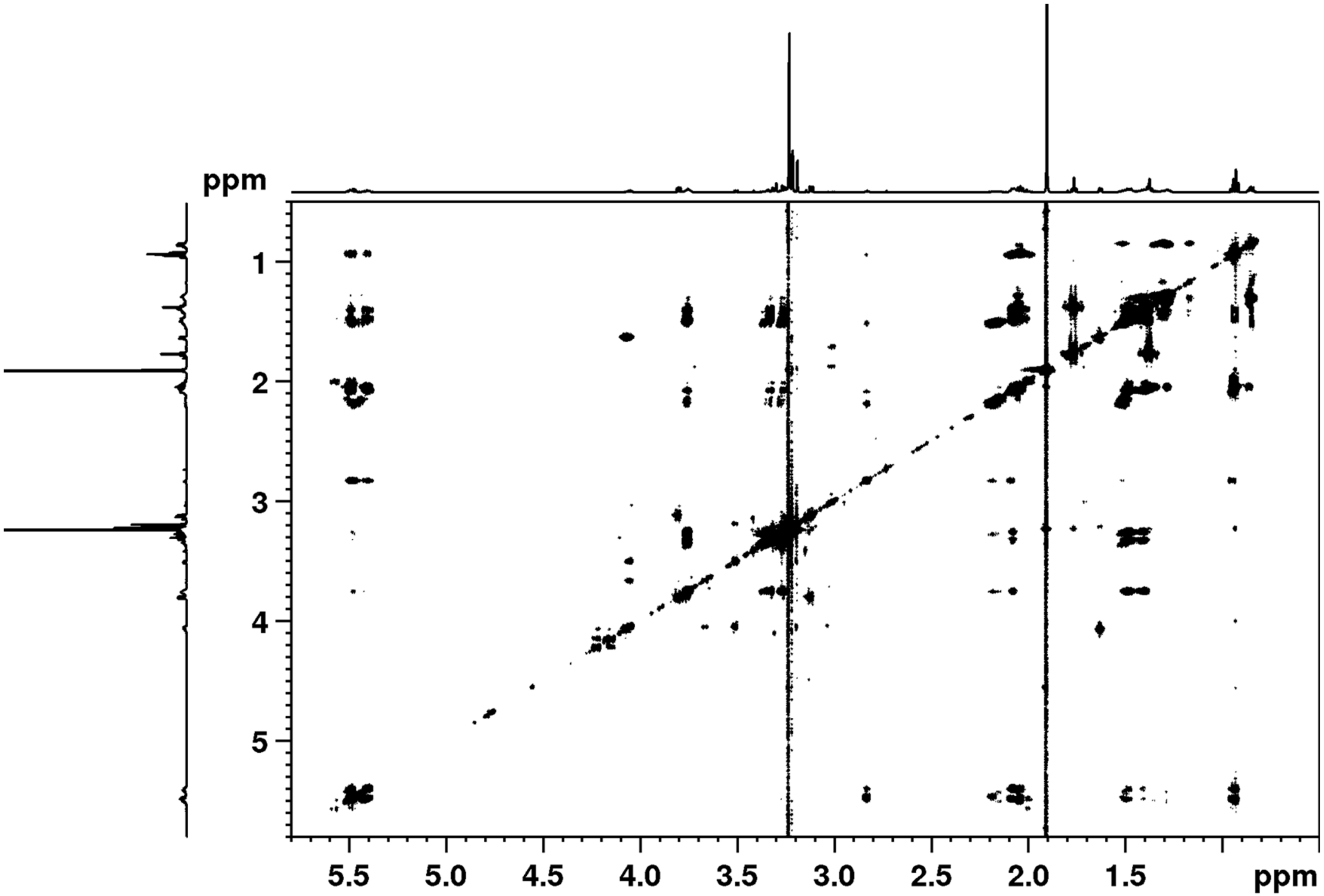

Figure S7. H,H-TOCSY NMR spectrum of carunculines.

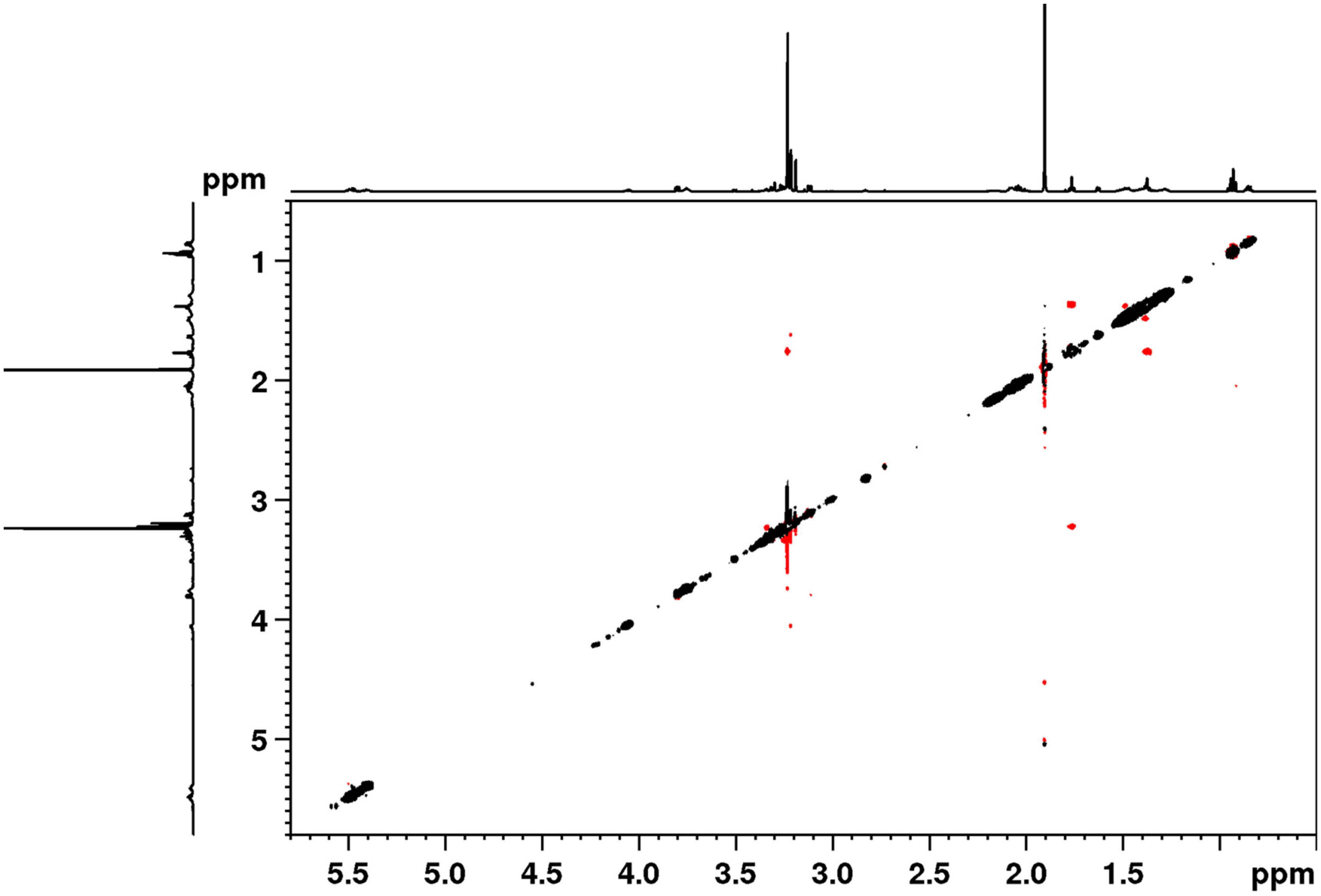

Figure S8. H,H-NOESY NMR spectrum of caruncolines. It clearly shows that protons at 1.76 ppm are those closer to the trimethylammonium group in A.

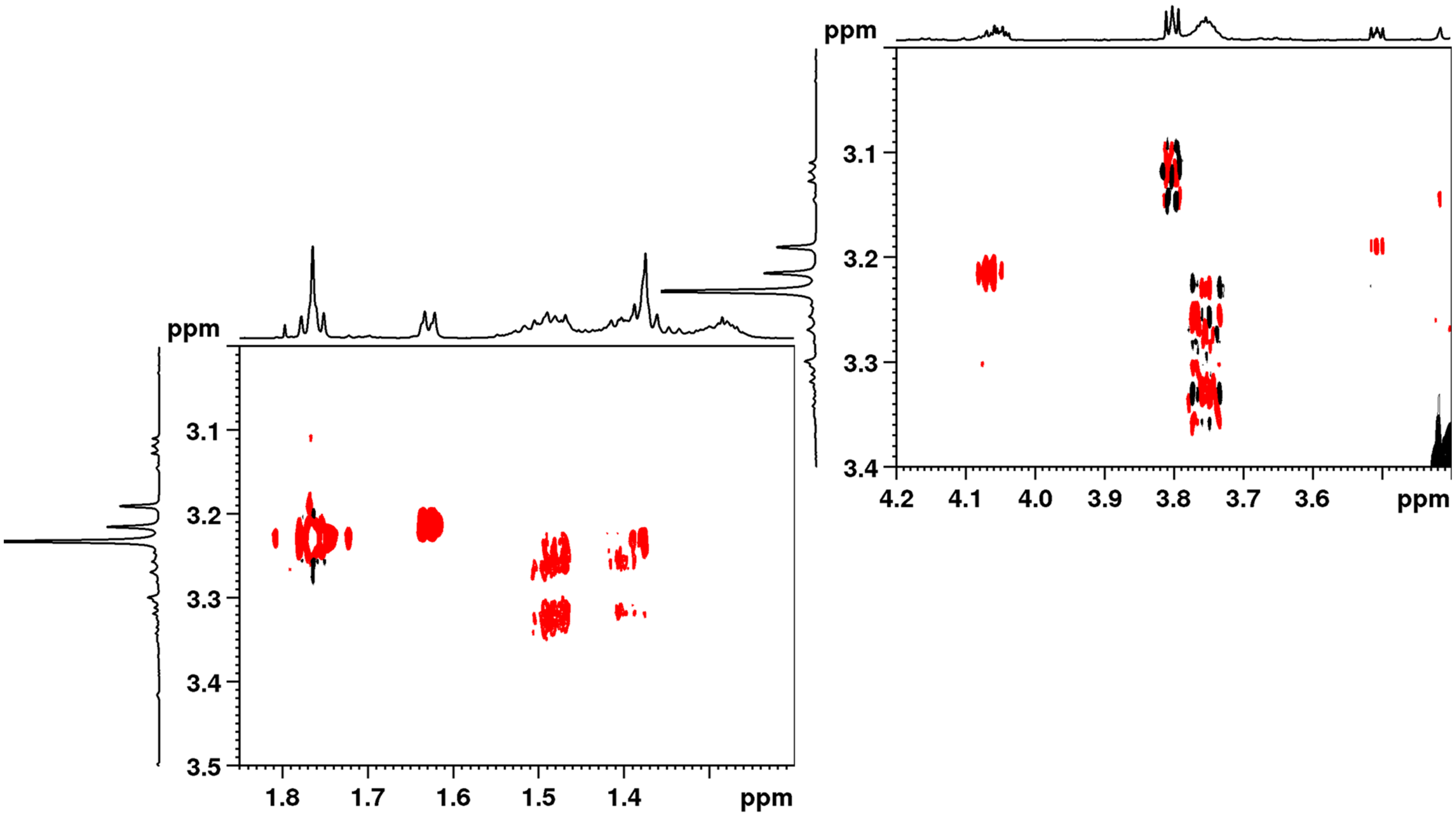

Figure S8 (continue). Enlarged regions of the H,H-NOESY NMR spectrum of carunculines. It clearly shows that protons at 1.76 ppm are those closer to the trimethylammonium group in A.

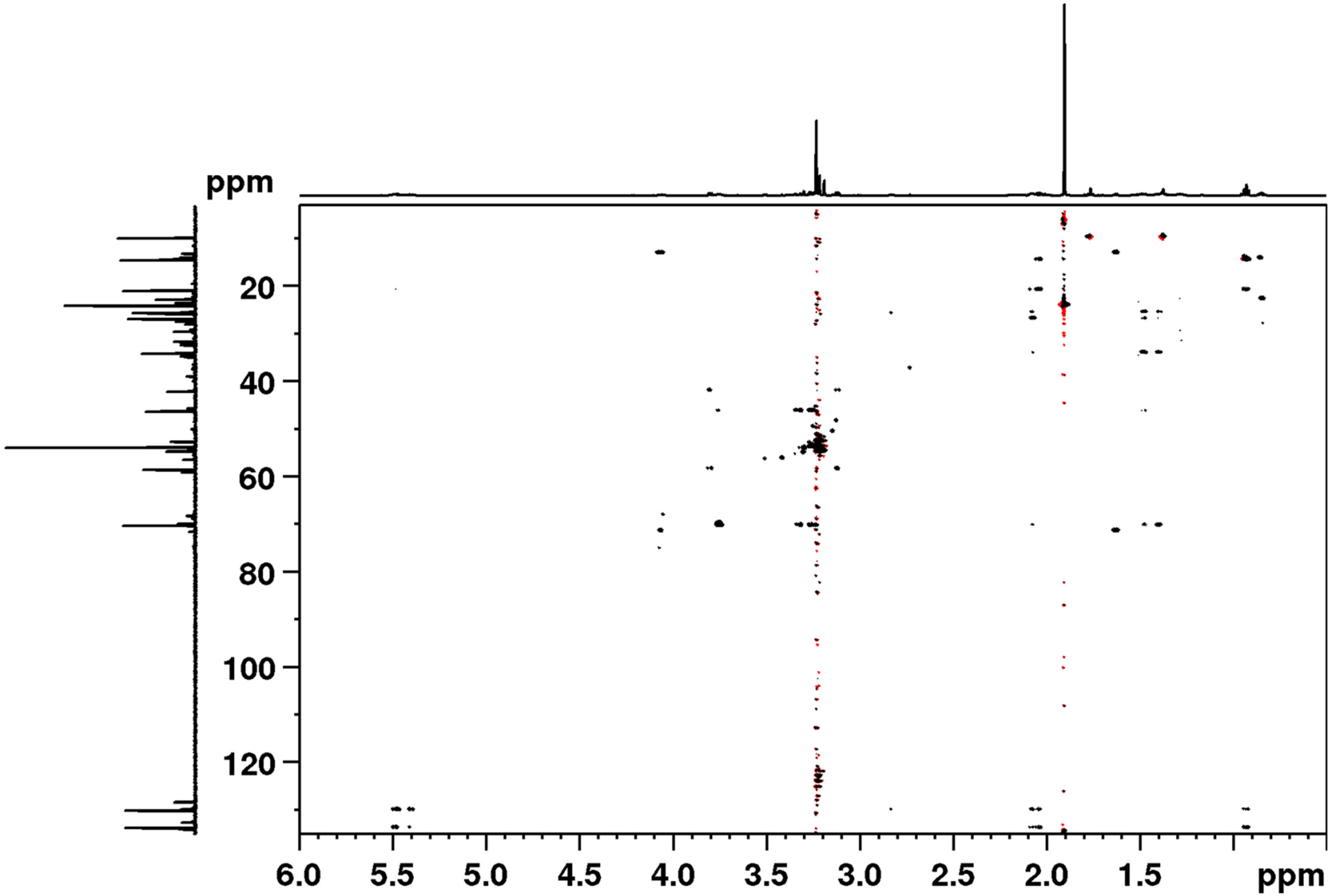

Figure S9. H,C-HSQC-TOCSY NMR spectrum of caruncullines.

Table S2. Relevant MS/MS fragments identified in carunculines (**1**,**3**,**5**,**8**) using HPLC-ESI/HRMS. Neutral loss (*m*) were obtained by difference between the *m/z* of each carunculine and the fragments. Hypothetical structures or formulae of the fragments lost were proposed.

Terminal ammonium portion (A)								
Carunculine **1** (*m/z* 283.2377)		Carunculine **3** (*m/z* 285.2535)		Carunculine **5** (*m/z* 309.2535)		Carunculine **8** (*m/z* 311.2693)		Proposed molecular structure / formula
MS/MS fragments		***MS/MS fragments***		***MS/MS fragments***		***MS/MS fragments***		
58.0659		***58.0659***		***58.0659***		***58.0659***		$CH_2=N(CH_3)_2^+$
60.0815		***60.0815***		***60.0815***		***60.0815***		$NH(CH_3)_3^+$
67.0549		***67.0549***		***67.0549***		***67.0549***		$C_5H_7^+$
84.0813		***84.0813***		***84.0813***		***84.0813***		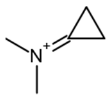
95.0859		***95.0859***		***95.0859***		***95.0859***		$C_7H_{11}^+$
98.0967		***98.0967***		***98.0968***		***98.0967***		
116.1071		***116.1071***		***116.1071***		***116.1071***		$C_6H_{14}NO^+$* 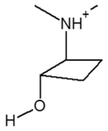
123.1168		***123.1168***		/		***123.1169***		$C_9H_{15}^+$
143.1177		***143.1178***		***143.1178***		***143.1178***		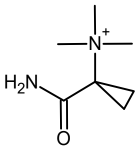
	neutral loss, *m*		neutral loss, *m*		neutral loss, *m*		neutral loss, *m*	
170.1536	113.084	***172.1693***	113.084	***196.1692***	113.0843	***198.1850***	113.0843	**-** 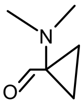
198.1850	85.053	***200.2006***	85.0529	***224.2007***	85.0528	***226.2163***	85.0530	**-** 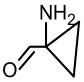
265.2270	18.011	***267.2426***	18.0107	***291.2426***	18.0109	***293.2582***	18.0111	**-** H_2O

*tentative structure

Table S3. Relevant MS/MS fragments identified in carunculines (**2**,**4**,**6**,**7**) using HPLC-ESI/HRMS. Neutral losses (*m*) were obtained by difference between the *m/z* of each carunculine and the fragments. Hypothetical structures or formulae of the fragments lost were proposed.

Terminal ammonium portion (B)								
Carunculine **2** (*m/z* 271.2377)		Carunculine **4** (*m/z* 273.2535)		Carunculine **6** (*m/z* 297.2535)		Carunculine **7** (*m/z* 299.2693)		Proposed molecular structure / formula
MS/MS fragments		***MS/MS fragments***		***MS/MS fragments***		***MS/MS fragments***		
58.0659		***58.0659***		***58.0659***		***58.0659***		$CH_2{=}N(CH_3)_2^+$
60.0815		***60.0815***		***60.0815***		***60.0815***		$NH(CH_3)_3^+$
67.0549		***67.0549***		***67.0549***		***67.0549***		$C_5H_7^+$
81.0704		***81.0704***		***81.0704***		***81.0704***		$C_6H_9^+$
95.0859		***95.0859***		***95.0859***		***95.0859***		$C_7H_{11}^+$
123.1168		***123.1168***		***123.1170***		***123.1169***		$C_9H_{15}^+$
	neutral loss, *m*		neutral loss, *m*		neutral loss, *m*		neutral loss, *m*	
140.1432	131.0945	***142.1589***	131.0946	***166.1591***	131.0944	***168.1746***	131.0947	- 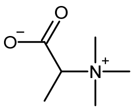
166.159	105.0787	***168.1745***	105.079	***192.1741***	105.0794	***194.1902***	105.0791	- H_2O - $N(CH_3)_3$ - CO
184.1694	87.0683	***186.1851***	87.0684	***210.1848***	87.0687	***212.2007***	87.0686	- $N(CH_3)_3$ - CO
194.1539	77.0838	***196.1694***	77.0841	***220.1649***	77.0886	***222.1851***	77.0842	- H_2O - $N(CH_3)_3$
253.2271	18.0106	***255.2427***	18.0108	***279.2428***	18.0107	***281.2583***	18.0110	- H_2O

Table S4. Carunculines (**1**–**8**) and their relative quantification in fireworm tissues based on peak area counts. The highest peak area value for each isomer was counted as 100% and the others were derived in proportion. DBW: Dorsal Body Wall; NO: notochaetae; NE: neurochaetae; PH: pharynx; GU: Gut; MS data: M^+: theoretical monoisotopic mass; i: isomer; RT is recorded at the apex of the peak; overall area: sum of isomer area. All peak areas derived from ion chromatograms obtained from 1 mg/ml samples.

Carunculines			*H. caruncluata* body parts				
			DBW	NO	NE	PH	GU
**1** (M+ 283.2380)		Exact mass	283.2380	283.2382	283.2382	283.2380	283.2379
	i1	Peak area	50.61%	56.15%	59.12%	7.93%	100.00%
		RT (min)	4.34	4.35	4.36	4.35	4.36
	i2	Peak area	35.56%	38.15%	36.81%	4.33%	100.00%
		RT (min)	4.50	4.50	4.50	4.51	4.49
		Overall area	47.20%	52.08%	54.16%	7.13%	100.00%
**2** (M+ 271.2380)		Exact mass	271.2381	271.2382	271.2381	271.2380	271.2379
	i1	Peak area	38.57%	32.07%	39.41%	5.67%	100.00%
		RT (min)	4.41	4.42	4.42	4.41	4.42
	i2	Peak area	35.40%	56.88%	100.00%	6.58%	97.82%
		RT (min)	4.30	4.30	4.31	4.31	4.30
	i3	Peak area	32.47%	26.89%	28.69%	3.94%	100.00%
		RT (min)	4.54	4.53	4.54	4.53	4.54
	i4	Peak area	21.60%	26.40%	36.60%	3.52%	100.00%
		RT (min)	4.13	4.14	4.14	4.13	4.14
		Overall area	34.81%	34.81%	47.51%	5.28%	100.00%
**3** (M+ 285.2536)		Exact mass	285.2537	285.2539	285.2538	285.254	285.2536
	i1	Peak area	44.67%	74.82%	90.63%	6.65%	100.00%
		RT (min)	5.11	5.11	5.11	5.10	5.10
	i2	Peak area	29.87%	61.62%	100%	5.50%	71.57%
		RT (min)	5.24	5.24	5.24	5.24	5.24
	i3	Peak area	30.05%	43.22%	60.49%	3.89%	100%
		RT (min)	5.01	5.01	5.02	5.01	5.02
		Overall area	38.46%	70.57%	100.00%	6.28%	91.06%
**4** (M+ 273.2535)		Exact mass	273.25380	273.25390	273.25370	273.25370	273.25360
	i1	Peak area	34.60%	52.78%	50.11%	4.85%	100.00%
		RT (min)	5.15	5.15	5.16	5.15	5.15
	i2	Peak area	31.83%	61.80%	44.57%	4.21%	100.00%
		RT (min)	5.07	5.06	5.07	5.07	5.06
	i3	Peak area	31.54%	41.91%	56.54%	5.51%	100.00%
		RT (min)	5.25	5.28	5.29	5.29	5.29
		Overall area	33.07%	50.39%	51.42%	4.99%	100.00%
**5** (M+ 309.2536)		Exact mass	309.25380	309.25410	309.25370	309.25380	309.25360
		Peak area	49.29%	82.56%	100.00%	7.34%	63.29%
		RT (min)	5.11	5.11	5.11	5.10	5.10
**6** (M+ 297.2535)		Exact mass	297.25360	297.25390	297.25370	297.25380	297.25380
	i1	Peak area	26.01%	35.18%	70.83%	3.17%	100.00%
		RT (min)	5.49	5.49	5.49	5.49	5.49
	i2	Peak area	25.83%	42.50%	85.42%	3.45%	100.00%
		RT (min)	5.35	5.35	5.34	5.35	5.34
		Overall area	25.99%	36.09%	72.40%	3.21%	100.00%
**7** (M+ 299.2693)		Exact mass	299.2693	299.2695	299.2693	299.2695	299.2693
	i1	Peak area	30.84%	100.00%	92.53%	4.82%	100%
		RT (min)	5.84	5.84	5.85	5.85	5.83
	i2	Peak area	29.50%	100%	96.27%	4.04%	95.65%
		RT (min)	5.91	5.91	5.91	5.92	5.91
	i3	Peak area	2.88%	5.95%	6.57%	0.46%	100%
		RT (min)	6.17	6.16	6.16	6.17	6.17
		Overall area	5.15%	13.69%	13.74%	0.80%	100.00%

**8** (M+ 311.2693)		Exact mass	311.2693	311.2693	311.2692	311.2694	311.2691
		Peak area	44.15%	61.13%	96.23%	7.37%	100.00%
		RT (min)	6.12	6.12	6.14	6.13	6.12

Table S5. Carunculines (**1**–**8**) and their relative quantification in marine invertebrates based on peak area counts. The highest peak area value for each isomer was counted as 100% and the others were derived in proportion. *A. vir*: *Anemonia viridis*; *Eise*: *Eisenia* sp.; *Peri.*: *Perinereis* sp.; *S. spa*: *Sabella spallanzanii*; *Sipu.*: *Sipunculus* sp.; *H. car*: *Hermodice carunculata*. MS data: M^+: theoretical monoisotopic mass; i: isomer; RT is recorded at the apex of the peak; overall area: sum of isomer area. All peak areas derived from ion chromatograms obtained from 1 mg/ml samples.

			**Marine invertebrates**					
**Carunculines**			***A. vir***	***Eise*. sp.**	***Peri*. sp.**	***S. spa***	***Sipu*. sp.**	***H. car***
**1** (M+ 283.2380)		Exact mass	283.2382	283.2382	283.2379	283.2383	283.2378	283.2380
	i1	Peak area	0.03%	0.18%	0.01%	0.17%	2.03%	100.00%
		RT (min)	4.37	4.59	4.37	4.37	4.37	4.28
	i2	Peak area	0.03%	0.09%	0.01%	0.08%	1.35%	100.00%
		RT (min)	4.50	4.75	4.50	4.46	4.51	4.48
		Overall area	0.03%	0.17%	0.01%	0.16%	1.92%	100.00%
**2** (M+ 271.2380)		Exact mass	271.2381	271.2382	271.2379	271.2384	271.2377	271.2380
	i1	Peak area	0.04%	0.18%	0.02%	0.10%	1.18%	100.00%
		RT (min)	4.42	4.65	4.43	4.43	4.44	4.39
	i2	Peak area	0.09%	0.23%	0.02%	0.13%	1.84%	100.00%
		RT (min)	4.32	4.55	4.31	4.31	4.32	4.23
	i3	Peak area	0.04%	0.11%	0.01%	0.06%	0.92%	100.00%
		RT (min)	4.58	4.81	4.58	4.53	4.53	4.56
	i4	Peak area	0.02%	0.08%	0.03%	0.06%	0.90%	100.00%
		RT (min)	4.15	4.38	4.19	4.14	4.15	4.12
		Overall area	0.04%	0.16%	0.02%	0.09%	1.15%	100.00%
**3** (M+ 285.2536)		Exact mass	285.2535	285.2539	285.2534	285.2541	285.2538	285.2536
	i1	Peak area	0.05%	0.13%	0.02%	0.11%	1.18%	100.00%
		RT (min)	5.11	5.30	5.12	5.12	5.12	5.08
	i2	Peak area	0.04%	0.12%	0.13%	0.12%	1.13%	100.00%
		RT (min)	5.25	5.43	5.25	5.26	5.25	5.23
	i3	Peak area	0.03%	0.07%	0.01%	0.06%	0.72%	100.00%
		RT (min)	5.02	5.22	5.04	5.01	5.01	5.01
		Overall area	0.04%	0.12%	0.01%	0.11%	1.11%	100.00%
**4** (M+ 273.2535)		Exact mass	273.2537	273.2540	273.2533	273.2540	273.2538	273.2537
	i1	Peak area	0.07%	0.15%	0.01%	0.09%	0.96%	100.00%
		RT (min)	5.16	5.34	5.16	5.16	5.16	5.14
	i2	Peak area	0.00%	0.00%	NF[1]	0.00%	0.00%	100.00%
		RT (min)	5.08	5.28	/	5.07	5.07	5.05
	i3	Peak area	0.07%	0.16%	0.02%	0.10%	0.88%	100.00%
		RT (min)	5.29	5.49	5.30	5.30	5.29	5.28
		Overall area	0.05%	0.12%	0.01%	0.09%	0.90%	100.00%
**5** (M+ 309.2536)		Exact mass	309.2536	309.2539	309.2534	309.2540	309.2535	309.2538
		Peak area	0.05%	0.13%	0.02%	0.11%	1.18%	100.00%
		RT (min)	5.11	5.30	5.12	5.12	5.12	5.08
**6** (M+ 297.2535)		Exact mass	297.2539	297.2538	297.2536	297.2541	297.2536	297.2538
	i1	Peak area	0.08%	0.26%	0.03%	0.16%	0.98%	100.00%
		RT (min)	5.49	5.67	5.50	5.50	5.48	5.49
	i2	Peak area	0.01%	0.02%	NF[1]	0.02%	0.19%	100.00%
		RT (min)	5.32	5.53	/	5.36	5.35	5.33
		Overall area	0.01%	0.04%	0.00%	0.03%	0.17%	100.00%
**7**		Exact mass	299.2694	299.2695	299.2691	299.2698	299.2694	299.2693

(M+ 299.2693)	i1	Peak area	0.06%	0.11%	0.01%	0.17%	1.15%	100.00%
		RT (min)	5.85	5.99	5.85	5.84	5.86	5.82
	i2	Peak area	0.09%	0.19%	0.03%	0.22%	1.45%	100.00%
		RT (min)	5.92	6.07	5.94	5.92	5.93	5.91
	i3	Peak area	0.08%	0.18%	0.03%	0.32%	100%	100.00%
		RT (min)	6.17	6.30	6.18	6.18	6.16	6.13
		Overall area	0.07%	0.16%	0.02%	0.25%	1.28%	100.00%
**8** (M+ 311.2693)		Exact mass	311.2693	311.2693	311.2690	311.2697	311.2692	311.2693
		Peak area	0.07%	0.17%	0.02%	0.36%	1.74%	100.00%
		RT (min)	6.12	6.27	6.13	6.14	6.13	6.07

[1]Not Found.

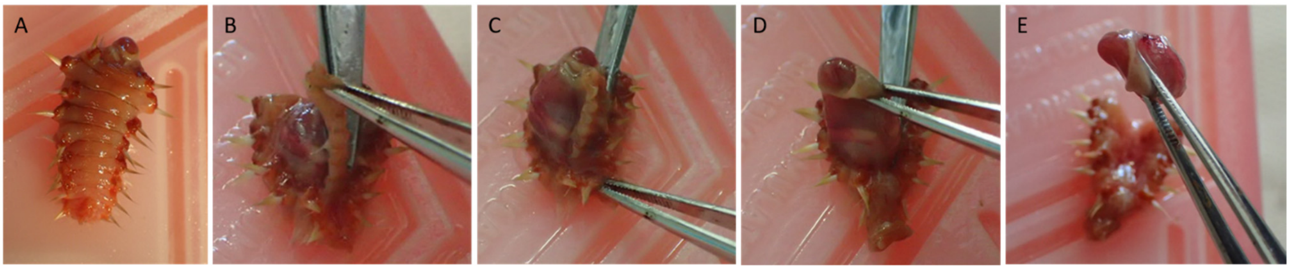

Figure S10 (A-E). Dissection of the pharynx. With a pair of sharp scissors, carefully make a cut at about chaetiger 15 on an anesthetized fireworm to remove the head including the whole pharynx. Place the head on an ice pack (A). With a pair of tweezers grab the dorsal body wall of the head in correspondence of the beheading cut. Using sharp scissors, cut laterally through the dorsal body wall on each side of the fireworm head toward the peristomium (A-C). Resect the two sets of dorsal and ventral muscles attached to the anterior and posterior portion of the pharynx until the mouth (D). Complete the dissection of the pharynx by cutting the buccal cavity close to the mouth (E). Transfer the pharynx in a glass bowl. Cover with parafilm and store at -18 ° C.

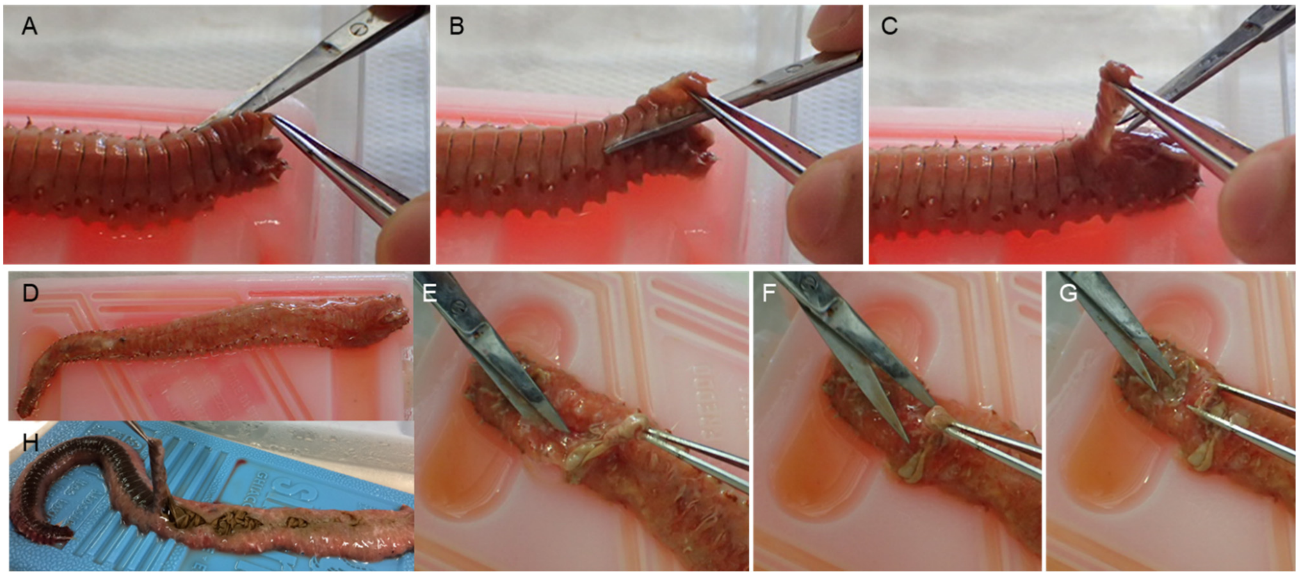

Figure S11 (A-H). Dissection of the dorsal body wall and gut. 6) With a pair of tweezers grab the dorsal body wall in correspondence of the beheading cut (A). Using sharp scissors, cut laterally through the dorsal body wall on each side of the fireworm up to the last 10-15 chaetigers (A-C). Once removed the dorsal body wall (D), grab the gut with tweezers starting from anterior portion of the body and pick it up to separate from the ventral body wall, using scissors if necessary (E-H). Dry the fragments on absorbent paper and transfer into a glass bowl. Cover with Parafilm and store at -18 ° C.

Table S6. Weight of the freeze-dried samples and acetone extracts obtained from the body parts of *H. carunculata* and from the marine invertebrate taxa examined.

Sample	Freeze-dried (mg)	Extract (mg)
**Tissues of *H. carunculata***		
Dorsal body wall	330.8	7.9
Notochaetae	1035.9	17.0
Neurochaetae	310.2	14.0
Pharynx	659.3	6.90
Gut	4144.6	1172
**Marine invertebrate sources**		
Hermodice carunculata (Annelida)	6860.0	106.0
Sipunculus sp. (Annelida)	4716.0	26.0
Perinereis sp. (Annelida)	12084.0	880.0
Sabella spallanzanii (Annelida)	8337.0	1290.0
Eisenia sp. (Annelida)	2854.0	167.0
Anemonia viridis (Cnidaria)	4667.0	339.0
Paracentrotus lividus (Echinodermata)	1227.0	79.0
Bolinus brandaris (Mollusca)	7395.0	171.0
Microcosmus sabatieri (Chordata)	7796.0	271.0
